# Integrin α5β1‐mediated multicellular crosstalk in the tumor microenvironment drives bladder cancer progression and reveals targetable vulnerabilities

**DOI:** 10.1002/imo2.70102

**Published:** 2026-06-23

**Authors:** Ting Liang, Wuwu Xu, Hu Fang, Lu Fu, Dashi Deng, Chenchen Li, Lisha Liu, Dongdong Tang, Huancheng Yang, Fang Liu, Xing Xiong, Guangzhi Li, Song Wu, Tao Tao

**Affiliations:** ^1^ College of Electronics and Information Engineering Shenzhen University Shenzhen China; ^2^ Department of Urological Surgery, The Third Affiliated Hospital of Shenzhen University (Shenzhen Luohu People's Hospital) Shenzhen University Shenzhen China; ^3^ Shenzhen Following Precision Medical Research Institute Luohu Hospital Group Shenzhen China; ^4^ Institute of Biomedical Data, South China Hospital, Medical School Shenzhen University Shenzhen China; ^5^ Institute of Pain, The Affiliated Hospital of Southwest Jiaotong University The Chengdu Third People's Hospital Chengdu China; ^6^ Department of Urology The First Affiliated Hospital of Zhengzhou University Zhengzhou China

**Keywords:** angiogenesis, bladder cancer, integrin α5β1, multicellular communication, single‐cell and spatial transcriptomics

## Abstract

Bladder cancer (BCa) progression is driven by complex interactions within the tumor microenvironment, yet the underlying multicellular communication networks remain poorly defined. Here, we integrated bulk, single‐cell, and spatial transcriptomic data with multiplexed immunofluorescence to construct a comprehensive cellular atlas across normal urothelium, precancerous lesions, and progressive BCa. We identified a stemness‐associated malignant epithelial population (Cluster 1_SDC1), a prognostically unfavorable subset of POSTN+ myofibroblastic cancer‐associated fibroblasts (mCAFs), and immunosuppressive monocytic myeloid‐derived suppressor cells (M‐MDSCs), all enriched in high‐grade tumors. Spatial mapping revealed a coordinated signaling cascade wherein POSTN+ mCAFs and M‐MDSCs co‐localize and engage integrin α5β1 on tip endothelial cells through FN1 and NAMPT, collectively driving angiogenesis. In turn, tip endothelial cells reinforce tumor stemness through downstream COL4A1/2–SDC1 signaling. Therapeutically, targeting integrin α5β1 with RGD peptide suppressed tumor growth and vascularization, while its combination with hydrogel‐based delivery and immune checkpoint blockade elicited broad‐spectrum antitumor responses. These findings uncover a spatially coordinated immune–stroma–tumor signaling cascade and establish integrin α5β1 as a central therapeutic target, providing a rationale for stroma‐directed combination strategies in progressive BCa.

## Highlights

- SDC1+ epithelial cells, POSTN+ mCAFs, and M‐MDSCs are enriched in high‐grade bladder cancer.
- Co‐localized mCAF‐FN1 and M‐MDSC‐NAMPT engage integrin α5β1 to drive angiogenesis.
- Tip endothelial cells then reinforce tumor stemness via COL4A1/2–SDC1 signaling.
- Targeting the integrin α5β1 cascade enhances immune checkpoint blockade efficacy.

## Graphical abstract

Integrating multiplexed immunofluorescence, animal models, and clinical samples, our single‐cell and spatial atlas maps tumor microenvironment evolution during bladder cancer (BCa) progression. We reveal that stemness‐associated tumor cells (SDC1+), POSTN+ myofibroblastic cancer‐associated fibroblasts (mCAFs), and immunosuppressive monocytic myeloid‐derived suppressor cells (M‐MDSCs) are enriched in high‐grade BCa. Mechanistically, mCAF‐derived FN1 and M‐MDSC‐derived NAMPT engage endothelial integrin α5β1 to drive angiogenesis. Subsequently, endothelial cells sustain tumor stemness via the COL4A1/2–SDC1 axis. Therapeutically, targeting integrin α5β1 synergizes with immune checkpoint blockade, highlighting a promising stroma‐directed strategy.

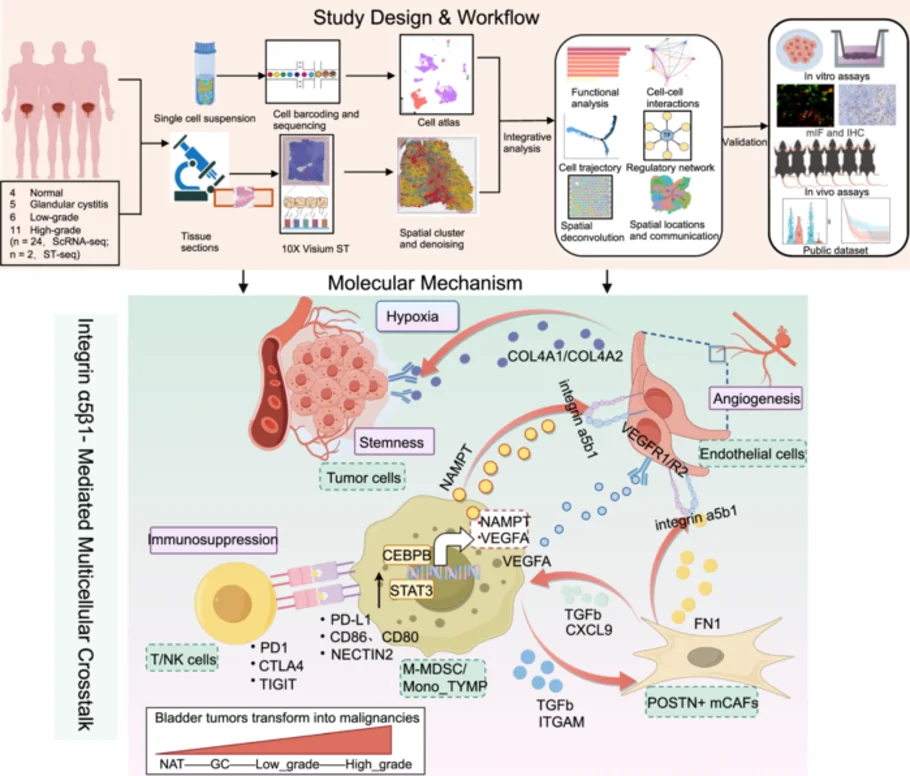

## 1 INTRODUCTION

Bladder cancer (BCa) is the tenth most common malignancy worldwide, with 573,000 new cases and 213,000 deaths reported in 2020 [1]. High‐grade BCa exhibits markedly higher recurrence, worse outcomes, and rapid progression to advanced stages without timely intervention compared with low‐grade tumors [2]. Although cystitis glandularis is recognized as a precursor lesion of BCa [3], the spatially resolved molecular events driving its microenvironment‐mediated malignant transformation remain poorly defined. Recent advances have identified novel regulators of BCa progression and treatment response. Liu et al. showed that the ADAR2/circ_0001005/miR‐200a‐3p/PD‐L1 axis modulates antitumor immunity [4], and Gao et al. reported that RSPO family members promote tumor progression via Wnt/β‐catenin activation [5]. However, standard therapies for advanced BCa—radical cystectomy and platinum‐based chemotherapy—still provide limited benefit [6, 7], and immune checkpoint inhibitors (ICIs) achieve response rates of only ~20% in cisplatin‐ineligible or refractory patients [8]. Notably, Zhang et al. recently demonstrated that Bavachalcone can overcome gemcitabine resistance by targeting transferrin receptor‐mediated iron metabolism, offering a potential therapeutic strategy and biomarkers for drug‐resistant BCa [9]. These emerging insights highlight the need to further elucidate the molecular and microenvironmental mechanisms underlying BCa progression and treatment resistance.

The shift toward precision oncology—including molecularly targeted therapies and immune checkpoint inhibitors—is fundamentally challenged by the ecological complexity of tumors, wherein the functional compartmentalization of malignant, stromal, and immune cells within the tumor microenvironment (TME) drives therapeutic resistance through spatiotemporal heterogeneity [8, 10]. Increasing evidence links the TME's cellular plasticity and the formation of immunosuppressive niches to poor clinical outcomes, particularly in immune‐excluded tumors where stromal barriers mediate drug resistance [11, 12]. Recent advances in spatial multiomics technologies, such as single‐cell RNA sequencing (scRNA‐seq) and spatial transcriptomics (ST‐seq), enable high‐resolution deconstruction of tumor ecosystems. These approaches uncover context‐specific cellular states, dynamic intercellular communication networks, and spatially resolved interactomes, offering critical insights for the development of microenvironment‐targeted therapeutic strategies [13, 14, 15].

Stromal cells, particularly endothelial cells and cancer‐associated fibroblasts (CAFs), have recently gained attention for their central roles in TME remodeling and tumor progression [16]. Using scRNA‐seq and ST‐seq, Pan et al. identified a distinct subset of APLN+ tip‐like endothelial cells (TipSI cells) associated with poor prognosis and potential responsiveness to bevacizumab‐induced vascular normalization [17]. CAFs, meanwhile, actively contribute to tumor initiation, recurrence, drug resistance, and vascular invasion in BCa [15, 18, 19, 20]. Their modulation of extracellular matrix dynamics and immune infiltration significantly influences tumor malignancy [21, 22]. Zhang et al. further classified fibroblast subtypes across multiple cancers, identifying MMP1+ fibroblasts as key mediators of immunosuppressive niches and resistance to immunotherapy [23]. Additionally, CAFs engage in extensive crosstalk with myeloid cells to shape the immunosuppressive TME [24]. For example, FAP+ CAFs promote tumor stemness by secreting IL‐6 and IL‐33 to regulate myeloid‐derived suppressor cells (MDSCs) in intrahepatic cholangiocarcinoma [25], while interactions between POSTN+ fibroblasts and SPP1+ macrophages drive the aggressiveness of head and neck squamous cell carcinoma [26].

Despite substantial progress, the intricate and dynamic interactions between tumor, stromal, and immune compartments in BCa malignant transformation remain inadequately understood. Decoding these complex cell–cell communications is crucial for unraveling how the TME is reshaped from precancerous lesions to malignant tumors. Such insights not only help understand TME remodeling but also provide a rationale for localized and sustained delivery strategies, such as hydrogel‐based systems, to modulate the TME with reduced systemic toxicity [27, 28]. To address this gap, we performed integrative scRNA‐seq and ST‐seq analyses across normal tissue, precancerous lesions (cystitis glandularis), low‐grade tumors, and high‐grade tumors. We aimed to characterize dynamic cellular and spatial alterations in the TME during BCa progression by delineating shifts in cellular composition—spanning malignant, stromal, and immune cells—and exploring how their spatial distribution and interactions evolve. This study offers critical insights into BCa malignant transformation and lays the foundation for developing rational combination strategies to optimize therapeutic efficacy through targeted interventions.

## 2 RESULTS

### 2.1 Identification of a distinct epithelial cell cluster linked to malignant transformation phenotypes

To delineate the cellular dynamics underlying BCa progression, we performed scRNA‐seq on 24 clinical samples, including normal urothelium (*n* = 4), cystitis glandularis (GC, *n* = 5), low‐grade BCa (*n* = 5), and high‐grade BCa (*n* = 10). After rigorous quality control, a total of 178,633 cells were retained for downstream analysis (Figure 1A, Figure S1A,B). To account for batch effects, the Harmony algorithm was applied for data integration. Dimensionality reduction and clustering, followed by annotation using canonical markers, revealed seven principal cell types: epithelial cells (*n* = 104,867), fibroblasts (*n* = 16,203), endothelial cells (*n* = 12,291), T/NK cells (*n* = 22,034), B cells (*n* = 9242), plasma cells (*n* = 8134), and myeloid cells (*n* = 5862) (Figure 1B–D, Figure S1C). Distinct heterogeneity was observed in the distribution and infiltration patterns of these cell types across different pathological stages. Notably, excluding cystitis samples, epithelial cells progressively accumulated with BCa malignancy, whereas the infiltration of T/NK cells exhibited a marked decline (Figure 1E, Figure S1D).

**Figure 1:**
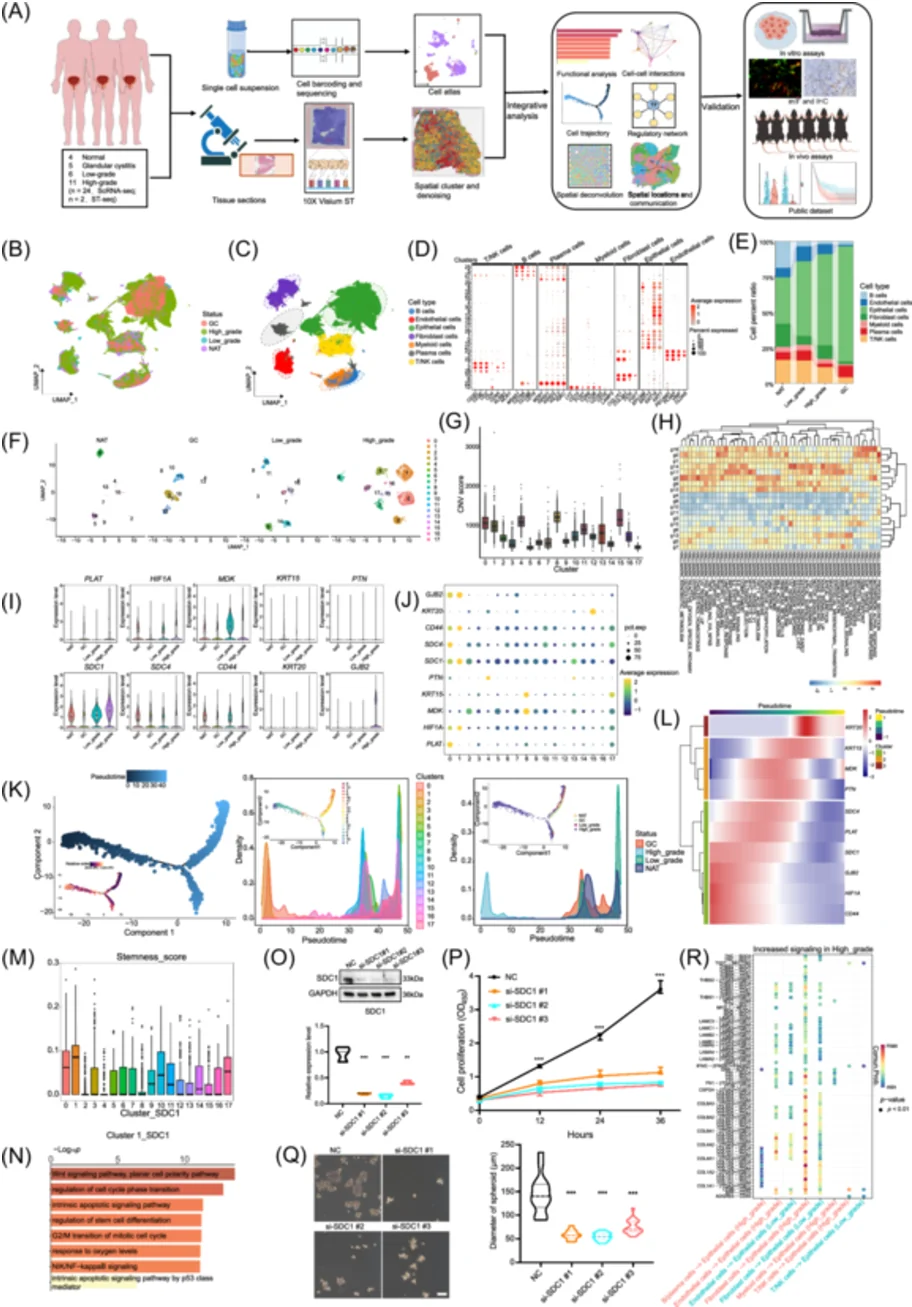
Single‐cell expression profiling identifies and characterizes malignant epithelial clusters in BCa. (A) Schematic overview of the study design, illustrating the sampling and analytical workflow. (B, C) UMAP visualization of 178,633 cells derived from 24 samples, encompassing NAT, GC, Low_grade, and High_grade tissues, identifying seven major cell types. (D) Expression profiles of established markers across the designated cell clusters. (E) Box plots illustrating the proportions of the seven major cell types across different pathological stages. (F) Re‐clustering of epithelial cells revealing distinct heterogeneity across NAT, GC, Low_Grade, and High_Grade tissues. (G) CNV score highlighting genomic instability across epithelial subclusters. (H) Pathway enrichment scores in different epithelial cell clusters (labeled as g0, g1, etc.), as determined by GSVA. (I) Violin plots illustrating the upregulation of malignancy‐associated genes in epithelial cells during BCa progression. (J) Dot plots displaying the expression levels of malignancy‐associated genes across re‐clustered epithelial cell groups. (K) Pseudotime developmental trajectory of epithelial cells inferred using Monocle2 and CytoTRACE2. (L) Heatmap displaying the dynamic expression patterns of malignancy‐associated genes along the pseudotime trajectory. (M) Stemness scores for different epithelial cell clusters calculated with AddModuleScore. (N) GO enrichment analysis highlighting key biological processes associated with Cluster 1_SDC1. (O) Validation of SDC1 knockdown efficiency by Western blot and qRT‐PCR in UMUC3 cells transfected with control or SDC1 siRNA. (P) CCK8 assay assessing the effect of SDC1 knockdown on UMUC3 cell proliferation. (Q) Representative images of colony formation in UMUC3 cells after SDC1 knockdown, showing colony size. Scale bar = 100 μm. Statistical analysis of the colony formation assay is shown on the right (*n* ≥ 10). (R) CellChat analysis illustrating altered intercellular signaling strengths received by epithelial cells in high‐grade tumors. Data are mean ± SD. Analyzed by Student's *t*‐test (O, Q) or two‐way ANOVA with Dunnett's test (P). **p < 0.01, ***p < 0.001 versus NC. BCa, bladder cancer; CCK8, Cell Counting Kit‐8; CNV, copy number variation; Comun. Prob., communication probability; GC, cystitis glandularis; GO, Gene Ontology; GSVA, Gene Set Variation Analysis; NAT, normal adjacent tissue; NC, negative control; pct.exp, percentage of expressed cells; qRT‐PCR, quantitative real‐time PCR; SDC1, syndecan 1; siRNA, small interfering RNA; UMAP, Uniform Manifold Approximation and Projection.

Given the pivotal role of epithelial cells in driving malignancy, we performed subclustering of epithelial populations across all stages, identifying 18 transcriptionally distinct subclusters (Figure 1F, Figure S1E). Strikingly, specific subclusters (Clusters 0, 1, 4, 8, and 15) were predominantly enriched in high‐grade BCa samples (Figure S1F). InferCNV analysis revealed prominent copy number variations (CNVs) in these clusters, suggesting elevated levels of malignancy (Figure 1G, Figure S1G,H). Differential gene expression profiling combined with Gene Set Variation Analysis (GSVA) demonstrated pronounced upregulation of genes involved in the hypoxia response (e.g., *PLAT*, *HIF1A*), glycolysis (*GJB2*) [29], and key signaling pathways governing cell proliferation, migration, and stemness, including *MDK* [30], *KRT20*, *SDC1/4* [31, 32], and *CD44*. Notably, these genes demonstrated progressively elevated expression with advancing malignancy (Figure 1H–J, Figure S1I–K). Immunohistochemistry (IHC) analysis corroborated these findings, with enhanced staining of HIF1A and SDC1 observed alongside BCa progression (Figure S1L).

To further explore epithelial cell plasticity, pseudotime trajectory analysis, utilizing Monocle2 and CytoTRACE2, unveiled a clear dedifferentiation trajectory in epithelial cells, with high‐grade tumor tissues predominantly occupying the dedifferentiated state. Remarkably, Cluster 1 was positioned at the root of this trajectory and exhibited the highest differentiation potential (Figure 1K). Along this continuum, key malignancy‐associated genes, including *GJB2*, *HIF1A*, *PLAT*, *MDK*, and *SDC1/4*, displayed substantial upregulation in high‐grade samples (Figure S1M,N). Intriguingly, *CD44* was enriched at the origin of the pseudotime trajectory, reflecting high differentiation potential and underscoring its role in stemness maintenance and malignant progression in high‐grade BCa (Figure 1L).

Among the identified subclusters, a distinct high‐grade BCa‐associated cluster characterized by elevated *SDC1* expression and stemness scores—termed Cluster 1_SDC1—was highlighted as a key malignant subpopulation (Figure 1M, Figure S1O). Gene Ontology (GO) enrichment analysis revealed significant activation of pathways involved in Wnt signaling, regulation of stem cell differentiation, G2/M cell cycle transition, and hypoxia response (Figure 1N). Correlation analysis further demonstrated a positive relationship between *SDC1* expression and stemness signatures (Figure S1P). Cellular functional assays, including spheroid formation, CCK8 assays, and wound‐healing assays, validated the role of SDC1 in promoting tumor cell stemness, proliferation, and migratory capacity (Figure 1O–Q, Figure S1Q). CellChat analysis revealed intensified stromal–epithelial crosstalk via collagen signaling in high‐grade BCa, highlighting the role of the TME in supporting malignancy (Figure 1R). Collectively, these results outline a dedifferentiation trajectory in BCa, highlighting SDC1+ epithelial cells (Cluster 1_SDC1) with stem‐like characteristics as key drivers of high‐grade malignant transformation.

### 2.2 Prognostic implications of POSTN+ mCAFs in high‐grade BCa

CAFs are recognized as key players in the unfavorable prognosis and progression of BCa [15, 19]. Based on distinct gene markers, we classified CAFs into two subtypes: inflammatory CAFs (iCAFs), characterized by *PDGFRA* and *CXCL14*, and myofibroblast‐like CAFs (mCAFs), marked by *RGS5* and *MMP11* (Figure 2A). Further gene expression analysis revealed nine CAF subpopulations (Figure 2B, Figure S2A,B), with POSTN+ mCAFs specifically enriched in high‐grade BCa tissues (Figure 2C). Periostin (POSTN), encoded by the *POSTN* gene, is a prominent extracellular matrix (ECM) protein implicated in numerous malignant biological processes, including cancer stem cell niche formation, angiogenesis, and metastasis [33, 34, 35]. Its elevated expression correlates with angiogenesis and poor prognosis in BCa (Figure S2C,D). In the TCGA‐BLCA cohort, POSTN+ mCAFs showed marked infiltration in high‐grade tumor tissues (Figure 2D), and their presence was strongly associated with poor patient outcomes (Figure 2E, Figure S2E). Moreover, this subgroup showed elevated activity in pathways related to epithelial‐to‐mesenchymal transition, ECM remodeling, collagen fibril organization, and type I interferon response, with angiogenesis being particularly pronounced. Notably, the enrichment of angiogenesis‐related pathways was most pronounced in fibroblasts within high‐grade tissue (Figure 2F,G Figure S2F–H). As BCa progresses, genes such as *MMP9*, *FN1*, and *COL6A2*, highly expressed in POSTN+ mCAFs, are progressively upregulated (Figure 2H,I, Figure S2I). MMP9 enhances angiogenesis by increasing VEGF bioavailability via MDSCs [36]. GO enrichment analysis highlighted these genes' involvement in myeloid cell differentiation, endoderm formation, and integrin binding (Figure 2J).

**Figure 2:**
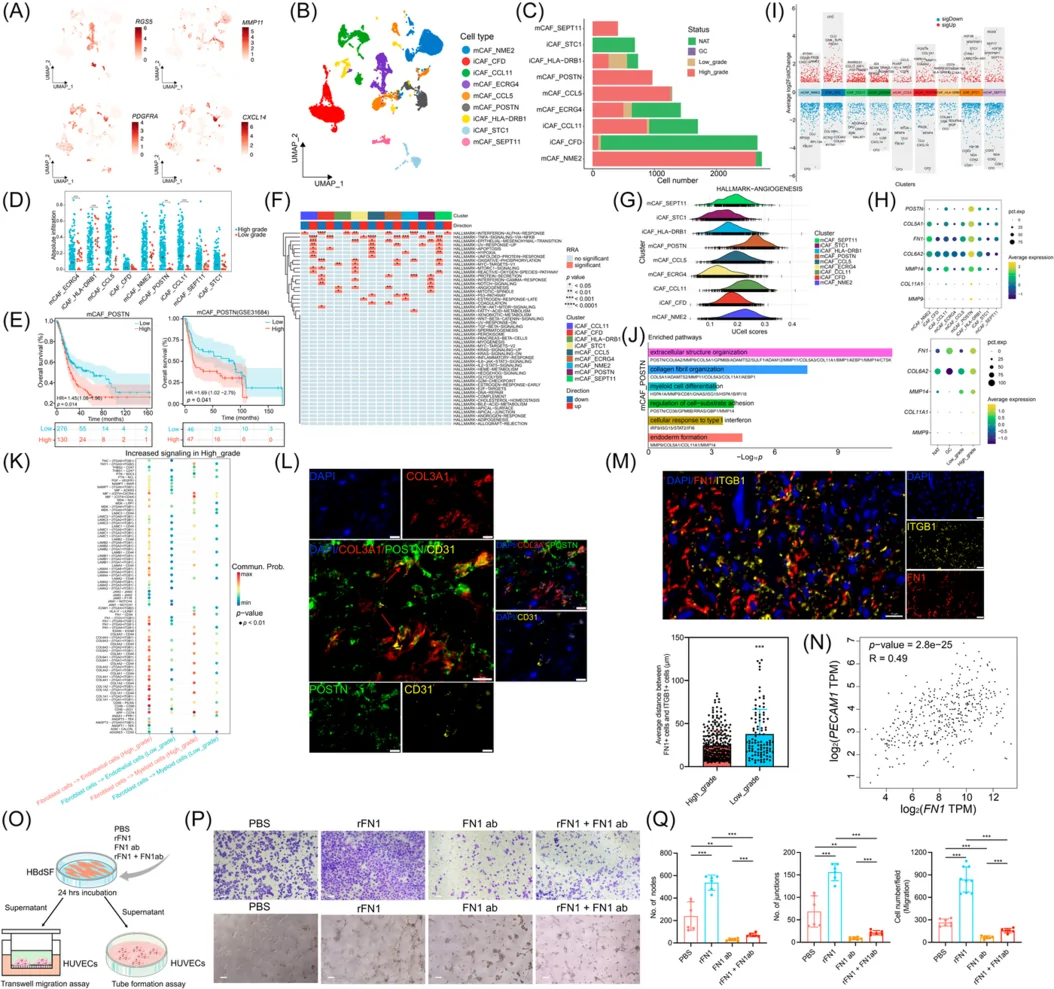
Identification of key cancer‐associated fibroblasts (CAFs) in high‐grade BCa. (A) UMAP plots displaying the expression of classical molecular markers for CAF subtypes. (B) UMAP plot illustrating the classification of distinct CAF subtypes. (C) Bar plots showing the cell count distribution of each CAF subtype across different sample types. (D) Box plots depicting the absolute infiltration levels of each CAF subtype in low‐grade (*n* = 21) and high‐grade (*n* = 381) tumor samples from TCGA‐BLCA. (E) Kaplan‐Meier overall survival curves stratified by POSTN+ mCAF infiltration in TCGA‐BLCA patients (left, *n* = 494) and in GSE31684 (right, *n* = 93). (F) Top 50 pathway enrichment scores per cell across different CAF subsets, analyzed by irGSEA. (G) Mountain plot highlighting the enrichment of angiogenesis gene sets across various CAF subtypes. (H) Dot plots displaying RNA expression of typical functional genes in fibroblasts from different CAF subtypes (top) and sample stages (bottom). (I) Volcano plot identifying the top five most significantly upregulated and downregulated genes among the nine CAF subtypes. (J) Bar chart showing GO term enrichment based on highly variable genes in POSTN+ mCAFs. (K) CellChat analysis illustrating signaling interaction strength from fibroblasts to endothelial and myeloid cells in high‐grade tumors. (L) Representative mIF images showing colocalization of POSTN+ mCAFs (POSTN + COL3A1+ double‐positive) and endothelial cells (CD31) in high‐grade bladder tumor samples. Scale bar = 10 μm. (M) mIF staining visualizing the spatial proximity between FN1+ cells and ITGB1+ cells. Quantification is shown below. Data are presented as mean ± SD. Scale bar = 10 μm. Statistical analysis by Student's *t*‐test; ***p < 0.001. (N) Spearman correlation analysis showing a positive association between *FN1* and *PECAM1* expression in TCGA‐BLCA. (O) Schematic of the conditioned medium transfer assays. (P) Representative images of HUVEC migration (top) and tube formation (bottom) using CM from HBdSFs treated with rFN1, FN1 ab, or their combination. Scale bar = 20 μm. (Q) Quantification of migrated cells, tube nodes, and junctions (n ≥ 6). Data are presented as mean ± SD. Statistical analysis by Student's *t*‐test; *p < 0.05, **p < 0.01, ***p < 0.001. CAF, cancer‐associated fibroblast; CM, conditioned medium; HBdSF, human bladder stromal fibroblast; HUVEC, human umbilical vein endothelial cell; mCAF, matrix CAF; mIF, multiplex immunofluorescence; TCGA‐BLCA, The Cancer Genome Atlas Bladder Cancer.

Cell‐cell communication analysis revealed that, in high‐grade BCa, POSTN+ mCAFs exhibit stronger interactions with endothelial and myeloid cells, primarily through the FN1–integrin α5β1 binding with endothelial cells (Figure 2K, Figure S2J,K). Multiplex immunofluorescence (mIF) staining confirmed the presence of POSTN+ mCAFs in BCa tissues, with higher fluorescence intensity observed in high‐grade tumors, particularly co‐localizing with endothelial cells (marked by CD31) (Figure 2L, Figure S2L). Additionally, FN1 and ITGB1 expression were elevated in high‐grade BCa, with FN1+ and ITGB1+ cells in close proximity (Figure 2M, Figure S2L). In the TCGA‐BLCA cohort, *FN1* expression was positively correlated with *CD31* expression (Figure 2N). To further validate the functional role of mCAF‐derived FN1 in mediating this stromal‐vascular crosstalk, we conducted supernatant transfer experiments (Figure 2O). We found that FN1 neutralization (FN1 ab) significantly impaired HUVEC migration and compromised tube formation networks. Conversely, recombinant FN1 (rFN1) treatment promoted these angiogenic phenotypes. Notably, this inductive effect was effectively abrogated by co‐treatment with the FN1 neutralizing antibody, confirming the specificity of FN1‐mediated stromal‐vascular crosstalk (Figure 2P,Q). Collectively, these findings highlight the enrichment of POSTN+ mCAFs in high‐grade BCa, linked to poor prognosis, and demonstrate their role in promoting tumor progression and angiogenesis specifically through FN1–integrin α5β1‐mediated endothelial crosstalk.

### 2.3 Preferential enrichment of MDSCs and their contribution to immune suppression and angiogenesis in high‐grade BCa

To explore the mechanisms through which the immune microenvironment facilitates BCa progression, we performed a comprehensive subtype annotation of myeloid cells, utilizing well‐established marker genes. This analysis led to the identification of 11 distinct subpopulations, including four macrophage clusters (defined by *C1QA*, *C1QB*, and *CD68*), two monocyte clusters (marked by *S100A9*, *CD14*, and *CST3*), two neutrophil clusters (distinguished by *CSF3R*, *IFITM2*, and *G0S2*), two dendritic cell clusters (cDC_LAMP3 and cDC_FCER1A), and mast cells (identified by *TPSAB1* and *KIT*) (Figure 3A,B, Figure S3A,B). A particularly noteworthy subpopulation, Mono_TYMP, characterized by high *TYMP* expression, was found to be significantly enriched in high‐grade BCa tissues (Figure 3C, Figure S3C). The expression levels of *TYMP* showed a robust correlation with angiogenesis (Figure S3D). Moreover, Mono_TYMP infiltration showed a positive correlation with POSTN+ mCAFs in the TCGA‐BLCA cohort (Figure 3D, Figure S3E) and was notably higher in high‐grade BCa tissues compared to low‐grade counterparts (Figure S3F). Hallmark pathway enrichment analysis revealed that Mono_TYMP is associated with pro‐tumorigenic programs, including inflammation, IL6–JAK–STAT3‐mediated immunosuppression, hypoxia, and angiogenesis (Figure 3E, Figure S3G). GO analysis further demonstrated enrichment of genes involved in lymphocyte suppression, myeloid differentiation, endothelial migration, and tube morphogenesis (Figure 3F). Trajectory analysis via Monocle2 positioned Mono_TYMP at an early stage of myeloid cell differentiation (Figure 3G), where it exhibited elevated expression of MDSC markers such as *CD11b*, *CD33*, and *S100A8/A9* (Figure S3H). Moreover, immune‐suppressive molecules, including *CD274*, *CD40*, *CD80*, *PDCD1LG2*, *NECTIN2*, and *TGFB1*, were found to be highly expressed in this subpopulation (Figure 3H). UCell scoring revealed that Mono_TYMP exhibited the highest MDSC score (Figure 3I), with this score progressively increasing during malignant progression in both BCa and lung adenocarcinoma (LUAD) (Figure 3J). Given its MDSC‐like characteristics, we classify Mono_TYMP as a monocytic MDSC (M‐MDSC) subtype and propose its critical role in driving BCa progression.

**Figure 3:**
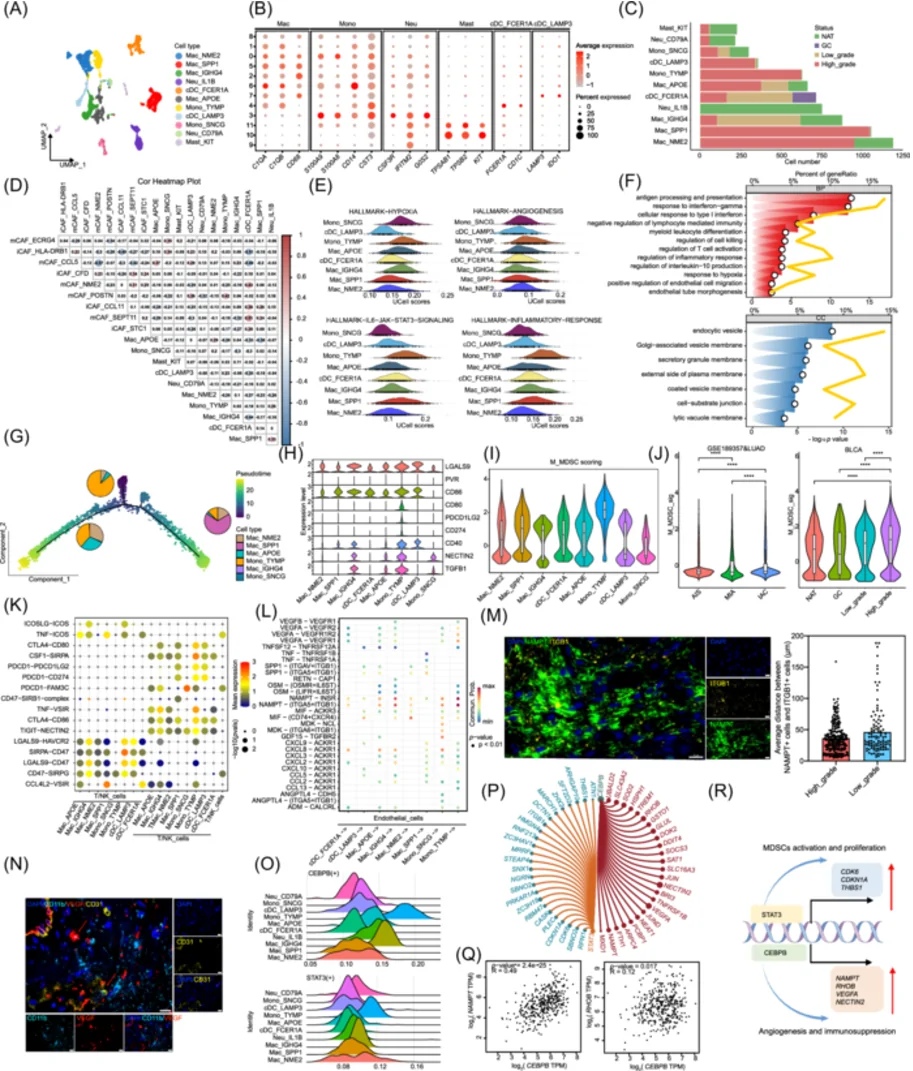
Transcriptional characterization and identification of myeloid‐derived suppressor cell (MDSC) subsets in high‐grade BCa. (A) UMAP plot illustrating the classification of myeloid cell subtypes. (B) Dot plots displaying the expression of common marker genes across each myeloid cell subset. (C) Bar plots illustrating the total cell numbers of each myeloid cell subset across various sample types. (D) Dot plots depicting Spearman correlation between CAFs and myeloid subtype infiltration in the TCGA‐BLCA cohort. The size of each dot is proportional to the absolute magnitude of the correlation coefficient, and the color intensity indicates the correlation value (red for positive, blue for negative). (E) Mountain plot illustrating the enrichment scores of selected Hallmark gene sets across different myeloid cell subtypes. (F) GO enrichment analysis highlighting BP and CC pathways based on highly variable genes in the Mono_TYMP subset. Bar length denotes enrichment significance (−log_10_ *p* value), while the yellow line represents the percent of geneRatio. (G) Pseudotime trajectory inferring the developmental evolution of myeloid cells. (H) Expression profiles of immune suppression‐related molecules across different myeloid cell subtypes. (I) Estimation of M‐MDSC‐related gene set scores across myeloid cell subtypes using AddModuleScore. (J) M‐MDSC scoring in the tumor progression of LUAD (GSE189357) and BCa. *p* values were calculated via two‐sided Wilcoxon rank‐sum test. (K) CellPhoneDB analysis of ligand–receptor interactions in coinhibitory signaling pathways between different myeloid cell subtypes and T/NK cells. (L) Bubble plot illustrating signaling pathway interactions from different myeloid cell subtypes to endothelial cells. (M) mIF staining visualizing the spatial proximity between NAMPT+ cells and ITGB1+ cells. Quantification is shown on the right. Data are presented as mean ± SD. *p* values were calculated via Student's *t*‐test. Scale bar = 10 μm. (N) Representative mIF images showing colocalization of VEGF‐expressing MDSCs and endothelial cells (CD31). Scale bar = 10 μm. (O) Mountain plot showing the activity of the STAT3 and CEBPB regulons. (P) Regulatory network plot illustrating the transcription factors (STAT3 and CEBPB) and their target genes. Node size is proportional to the node degree (number of connections). Node and edge colors represent the specific transcription factor regulons (STAT3 network in red, CEBPB network in blue). (Q) Spearman correlation analysis showing the association between the expression of *CEBPB* and *NAMPT*, as well as the angiogenesis‐related gene *RHOB*, in the TCGA‐BLCA cohort. (R) Schematic diagram of the regulatory network. Black arrows indicate transcriptional activation of target genes by STAT3 and CEBPB. Red upward arrows denote the upregulation of these genes. *p < 0.05; **p < 0.01; ***p < 0.001. BP, biological process; CC, cellular component; LUAD, lung adenocarcinoma; MDSC, myeloid‐derived suppressor cell; M‐MDSC, monocytic MDSC.

To further elucidate the functional role of Mono_TYMP, we applied advanced cell–cell communication analysis to dissect its interactions within the TME. A significant enhancement of cellular crosstalk was observed in high‐grade tumors, particularly between myeloid and T/NK cells, as well as between myeloid and endothelial cells (Figure S3I). CellPhoneDB analysis revealed that Mono_TYMP suppresses T/NK cell activity through immunoinhibitory ligand–receptor interactions, including CD274–PDCD1, CD80–CTLA4, CD86–CTLA4 and NECTIN2–TIGIT (Figure 3K). In parallel, CellChat uncovered strong communication between Mono_TYMP and endothelial cells, primarily via NAMPT–(ITGA5 + ITGB1) and VEGFA–VEGFR1/2 signaling axes (Figure 3L). Pseudotime analysis demonstrated early upregulation of key immunoregulatory and pro‐angiogenic genes—*TGFB1*, *CD274*, *NAMPT*, and *VEGFA*—during Mono_TYMP differentiation (Figure S3J,K). Consistently, mIF analysis showed that NAMPT+ cells were closely associated with ITGB1+ cells in high‐grade tumors, indicating upregulation of NAMPT–ITGB1 signaling during tumor progression (Figure 3M, Figure S3L). Furthermore, VEGFA‐expressing MDSCs colocalize with endothelial cells, with enhanced signaling in high‐grade BCa (Figure 3N, Figure S3M).

To uncover upstream regulatory mechanisms, we performed SCENIC analysis, which revealed pronounced STAT3 activation in the Mono_TYMP cluster, driving MDSC activation and proliferation [36, 37], and closely linked to the regulation of cell cycle‐related genes, including *CDK6* and *CDKN1A* (Figure 3O,P, Figure S3N,O). CEBPB also emerged as a crucial regulator within Mono_TYMP, modulating pro‐angiogenic and immunosuppressive factors, including *NAMPT*, *RHOB*, *VEGFA*, and *NECTIN2* (Figure 3O,P, Figure S3N). Correlation analysis in the TCGA cohort further corroborated a positive association between *CEBPB* expression and factors like *NAMPT* and *RHOB* (Figure 3Q). To experimentally validate these regulatory interactions, we knocked down *CEBPB* in M‐MDSCs derived from human PBMCs (Figure S4A,B). Consistent with our transcriptional predictions, *CEBPB* silencing significantly downregulated the expression of downstream targets, including *NAMPT*, *RHOB*, and *NECTIN2* (Figure S4C). Furthermore, functional assays demonstrated that conditioned medium from *CEBPB*‐deficient M‐MDSCs significantly impaired HUVEC migration and tube formation capabilities compared to controls, confirming that CEBPB is essential for the pro‐angiogenic function of these cells (Figure S4D–G). Collectively, these findings position STAT3 and CEBPB as core regulators of the Mono_TYMP phenotype and identify Mono_TYMP as a key M‐MDSC subset in high‐grade BCa, promoting tumor progression through coordinated immune suppression and angiogenic signaling (Figure 3R).

### 2.4 Crosstalk between tip_vECs and POSTN + mCAFs/MDSCs through integrin α5β1 fosters angiogenesis

Given the pro‐angiogenic potential of POSTN+ mCAFs and Mono_TYMP cells, we next investigated functional alterations in endothelial cells during BCa progression. As the disease advances, the expression of key neovascularization‐associated genes—such as *VWF*, *FN1*, *COL4A2*, *ANGPT2*, and *COX2*—increases progressively, indicating enhanced angiogenic activity (Figure S5A–D). Unsupervised clustering identified nine distinct endothelial subpopulations (Figure 4A), with most cells expressing *VWF*, a canonical marker of vascular endothelial cells (vECs), and a minority expressing *PROX1*, characteristic of lymphatic endothelial cells (Figure S5E). Based on endothelial subtyping across multiple cancer types as described by Zhang et al. [38], clusters En2/3/4/7 were annotated as Tip‐like vECs (Tip_vECs), marked by high expression of *COL4A1*, *KDR*, and *ESM1* (Figure 4B). These Tip_vECs were predominantly enriched in high‐grade BCa (Figure 4C) and exhibited elevated expression of pro‐angiogenic genes such as *ANGPT2*, *COL4A2*, and *COX2* (Figure S5E). GO analysis revealed significant enrichment of angiogenesis‐related pathways in Tip_vECs, including ATP synthesis‐coupled metabolism, ameboid‐type cell migration, hypoxia response, and endothelial proliferation and migration (Figure 4D). Hallmark gene set analysis further confirmed robust activation of angiogenesis, alongside notable upregulation of Hedgehog signaling, a pathway linked to stem‐like phenotypes [39] (Figure 4E, Figure S5F). Importantly, PI3K signaling—a key regulator of COX2 and tumor angiogenesis [40, 41]—was substantially activated in Tip_vECs (Figure 4F), underscoring its central role in driving endothelial pro‐angiogenic programs.

**Figure 4:**
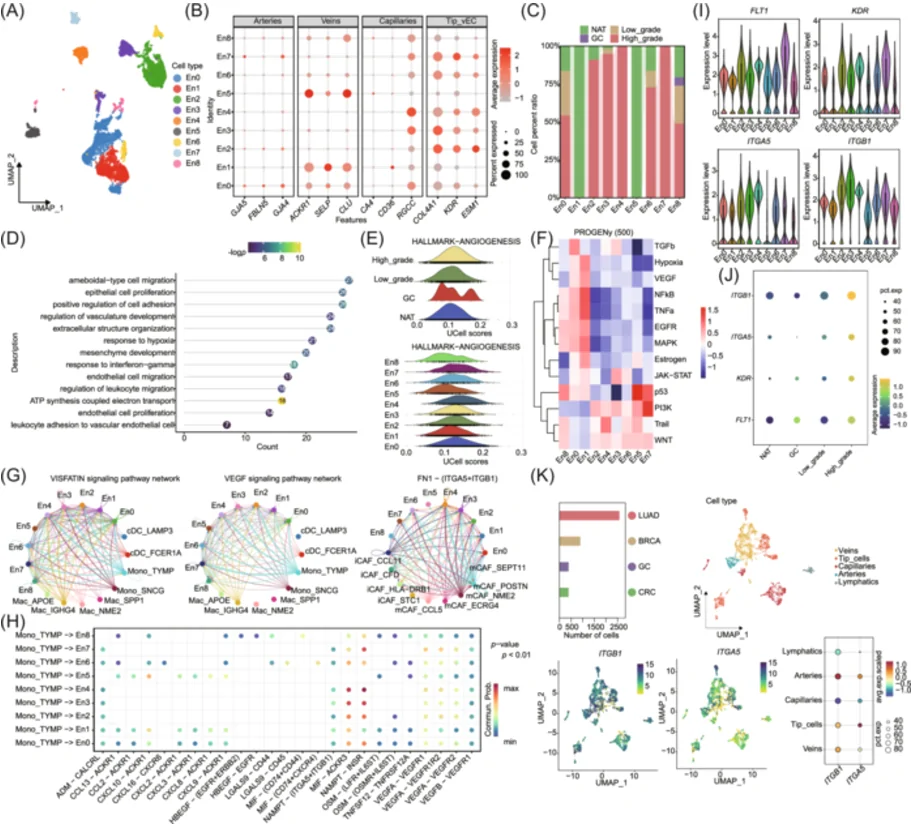
Characterization of vascular‐promoting phenotypes in endothelial cell subsets within high‐grade tumor tissues. (A) UMAP plot illustrating the classification of endothelial cell subclusters. (B) Dot plots displaying the expression of typical marker genes across different endothelial cell subtypes. (C) Sankey plot showing the variation in proportions of endothelial cell subclusters across sample types. (D) GO pathway enrichment analysis based on highly variable genes in specific endothelial subclusters (En2/3/4/7). (E) Activity of Hallmark angiogenesis pathways across different sample types (top) and endothelial cell subclusters (bottom). (F) Heatmap showing the differential activation of PROGENy pathways across endothelial cell subclusters. (G) Circle plots illustrating the cell–cell interaction strength between endothelial cell subclusters and myeloid cell subtypes in VISFATIN and VEGF signaling pathways, as well as between endothelial cell subclusters and CAF subtypes in the FN1 signaling pathway. (H) Bubble plot illustrating ligand–receptor interaction strength between the Mono_TYMP subtype and endothelial cell subclusters. Expression profiles of *FLT1*, *KDR*, *ITGA5*, and *ITGB1* across different endothelial cell subclusters (I) and different sample types (J). (K) Endothelial cell characteristics across single‐cell datasets from multiple cancer types. VEGF, vascular endothelial growth factor.

To further investigate the potential involvement of Tip_vECs in mediating crosstalk between POSTN+ mCAFs and Mono_TYMP cells, we employed CellChat to delineate the intercellular communication landscape among endothelial, myeloid, and fibroblast subpopulations in the TME. Remarkably, a prominent interaction was observed between MDSCs (Mono_TYMP) and Tip_vECs (En2/3/4/7), primarily mediated through the Visfatin (NAMPT_ITGA5/ITGB1) and VEGF (VEGFA_VEGFR1/R2) signaling axes (Figure 4G,H, Figure S5G,H). Additionally, POSTN+ mCAFs exhibited the most frequent interactions with Tip_vECs, with FN1 secretion driving the binding to ITGA5/ITGB1 on Tip_vECs, thereby promoting angiogenesis (Figure 4G, Figure S5G,I,J). Receptors for VEGF (FLT1 and KDR) and integrin α5β1 (ITGA5 and ITGB1) were highly expressed in Tip_vECs, with peak levels in endothelial cells from high‐grade tumors (Figure 4I,J). Moreover, integrin α5β1 expression exhibited a significant positive correlation with CD31, a well‐established endothelial marker (Figure S5K). Intriguingly, Tip_vECs with high integrin α5β1 expression were also identified across various tumor types, including LUAD, breast cancer (BRCA), gastric cancer (GC), and colorectal cancer (CRC) (Figure 4K), suggesting integrin α5β1 as a potential pan‐cancer target for anti‐angiogenic therapy. In conclusion, these findings delineate a pro‐angiogenic interaction network centered on integrin α5β1 and suggest its potential as a broadly conserved therapeutic target in cancer.

### 2.5 Spatial architecture and multicellular signaling cascades in high‐grade BCa

To elucidate spatial dynamics, we analyzed ST data from high‐ and low‐grade BCa patients, examining the spatial architecture and distribution of scRNA‐seq‐identified cell subsets, as well as the proportions and functional signatures of distinct tumor regions (Figure 5A–D, Figure S6A). Using spatially enriched gene expression and GO enrichment analysis, we classified regions 0, 1, 2, and 4 as tumor‐dominant zones, marked by the presence of hallmark genes such as *MMP7* and *KRT13*, while regions 3 and 5 were identified as stromal areas, distinguished by markers like *COL1A1*, *COL3A1*, and *VWF* (Figure S6B). Region 0 exhibited a significantly higher proportion in high‐grade BCa (Figure 5B) and was characterized by tumor‐promoting features, including regulation of epithelial differentiation, proliferation, response to drugs, glycolytic process, hypoxic responses, and stemness‐related signatures (Figure 5C, Figure S6E,F). In contrast, regions 3 and 5 showed stronger activation of pathways related to ECM remodeling, wound healing, ameboidal‐type cell migration, and angiogenesis in high‐grade compared to low‐grade tumor tissue (Figure 5D, Figure S6C,D,G).

**Figure 5:**
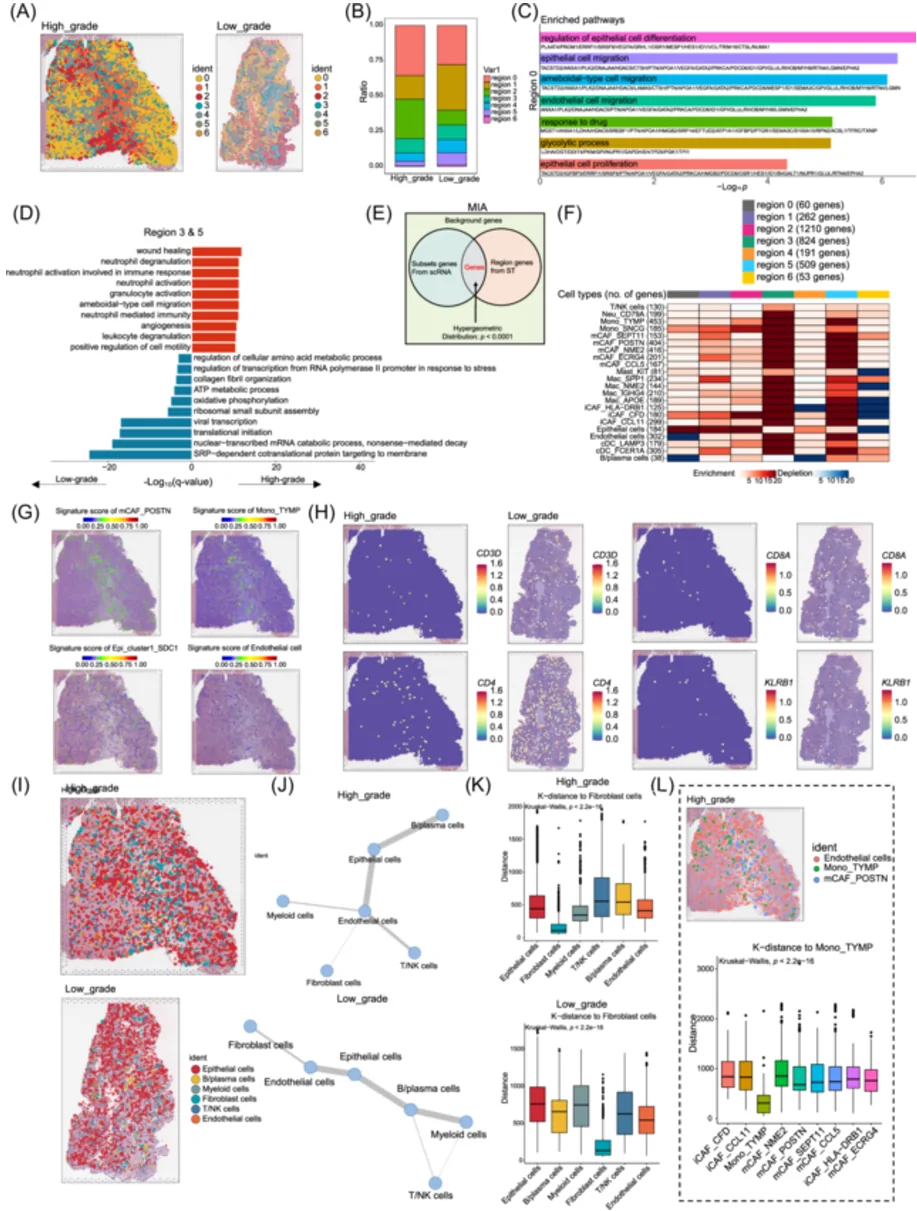
Spatial heterogeneity of the tumor microenvironment (TME) in low‐ versus high‐grade bladder tumors. (A, B) Spatial localization and relative composition of distinct regions in low‐ and high‐grade tumors, illustrating region‐specific organization of the TME. (C) GO enrichment analysis characterizing the distinct biological functions of Region 0. (D) GSVA highlighting differential pathway activities in Regions 3 and 5 between low‐grade and high‐grade tumors. (E) Schematic overview of the MIA workflow. (F) Heatmaps illustrating the spatial enrichment of scRNA‐seq‐defined cell subgroups within ST regions. (G) Spatial feature plots visualizing the distribution of mCAF_POSTN, Mono_TYMP, Epi_cluster1_SDC1, and endothelial cells in high‐grade tissue sections. (H) Spatial feature plots showing the gene expression of *CD3D*, *CD8A*, *CD4*, and *KLRB1* in low‐grade and high‐grade tissue sections. (I) High‐resolution spatial mapping of single‐cell phenotypes onto tissue architecture. (J) Spatial colocalization analysis of various cell types across high‐grade (top) and low‐grade (bottom) tumor tissues. Connecting line thickness indicates the strength of spatial colocalization. (K) Proximity analysis quantifying the spatial distance between fibroblasts and other cell types in high‐grade (top) and low‐grade (bottom) tumor tissues. (L) Spatial colocalization of mCAF_POSTN, Mono_TYMP, and endothelial cells (top) and spatial distance between Mono_TYMP and various CAF subtypes (bottom). MIA, multimodal intersection analysis; scRNA‐seq, single‐cell RNA sequencing; ST, spatial transcriptomics; TME, tumor microenvironment.

To further investigate the spatial distribution of specific cell subsets, we integrated scRNA‐seq and ST data using multimodal intersection analysis (MIA) (Figure 5E). Tumor cells predominantly localized to regions 0, 1, 2, and 4, which exhibited minimal T/NK cell infiltration. In contrast, stromal regions 3 and 5 were enriched for fibroblasts and myeloid cells—particularly POSTN+ mCAFs, Mono_TYMP cells, and endothelial cells—and displayed significantly higher levels of T/NK cells (Figure 5F, Figure S7A). In situ expression profiling of immune markers (*CD3D*, *CD4*, *CD8A*, and *KLRB1*), along with spatial signature scores of specific subclusters (POSTN+ mCAFs, Mono_TYMP, Epi_cluster1_SDC1, and endothelial cells), suggested that POSTN+ mCAFs and Mono_TYMP cells collaboratively orchestrate a spatially resolved immunoregulatory network that restricts T/NK cell infiltration within tumor‐enriched regions (Figure 5G,H).

To further delineate spatial interactions, we applied CellTrek to project single‐cell transcriptomes onto ST slices, revealing significantly closer spatial proximity between myeloid cells and fibroblasts in high‐grade tumors compared to low‐grade lesions (Figure 5I–K). Notably, POSTN+ mCAFs were frequently juxtaposed with Mono_TYMP cells and co‐localized with endothelial populations (Figure 5L, Figure S7B). Spatial communication analysis via CellChat identified region 3 as a major source of ligands—including FN1, NAMPT, and VEGFA—that target integrin α5β1 (ITGA5/ITGB1) and VEGFR1/2 receptors in region 5 (Figure 6A,B, Figure S7C–F). In situ visualization further confirmed co‐localization of key ligand–receptor pairs, such as FN1_(ITGA5 + ITGB1), NAMPT_(ITGA5 + ITGB1), and VEGFA_VEGFR1/R2 (Figure S7D–F,G,H). Additionally, region 5, enriched in vascular endothelial markers such as *VWF* and *COL4A1/2*, displayed robust activation of the COL4A–SDC1 signaling cascade directed toward the adjacent stemness‐associated tumor compartment (region 0) (Figure 6C, Figure S7I). Functional assays demonstrated that exogenous collagen IV treatment promoted tumor stemness, whereas this effect was significantly abrogated in SDC1‐knockdown cells, verifying the essentiality of the COL4A–SDC1 axis (Figure S8A,B). Furthermore, region 5 was spatially characterized by high stemness scores (Figure S6F), suggesting that COL4A1/2‐enriched endothelial cells promote neovascularization in hypoxic niches while concurrently reinforcing tumor stemness [39, 42]. These findings highlight COL4A1/2 as a pivotal endothelial‐derived matrix component linking angiogenic activation to stemness maintenance.

**Figure 6:**
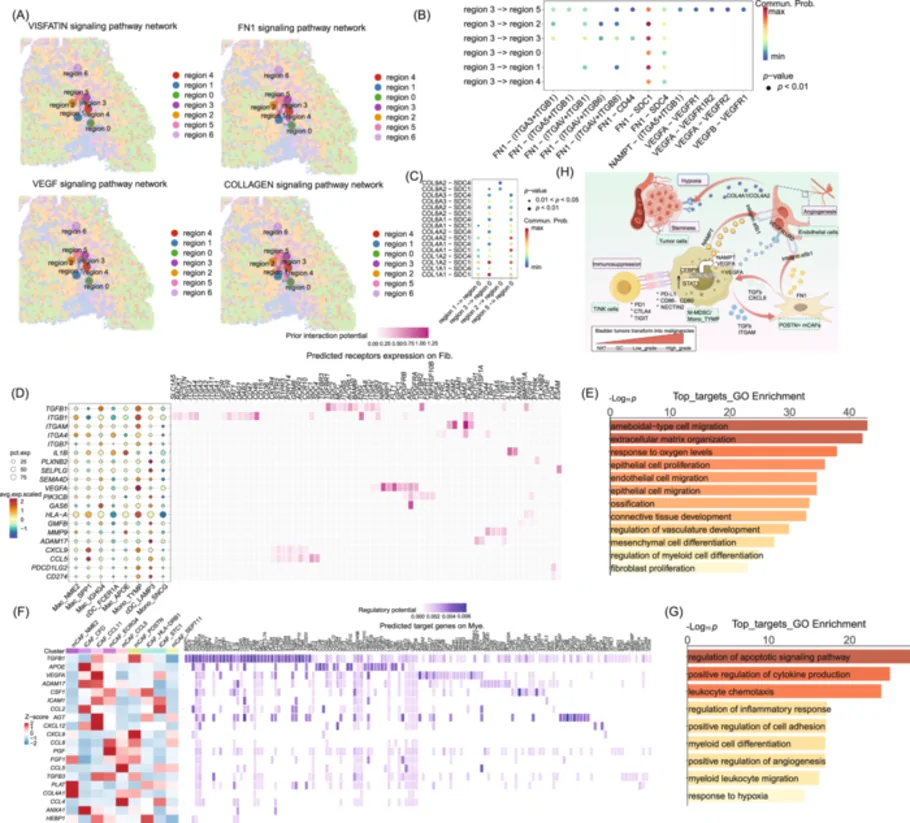
Spatial communication networks across various regions. (A) Spatial communication networks for the VISFATIN, FN1, VEGF, and COLLAGEN signaling pathways across distinct regions. (B) Bubble plot showing signaling interactions of VISFATIN, FN1, and VEGF pathways originating from Region 3 to other spatial regions. (C) Bubble plot illustrating COLLAGEN signaling pathway interactions directed from various regions to Region 0. (D) Heatmap depicting regulatory interactions from various myeloid subtypes to fibroblasts by NicheNet analysis. (E) GO pathway enrichment analysis for predicted target genes expressed in fibroblasts. (F) Heatmap depicting regulatory interactions from distinct CAF subsets to myeloid cells by NicheNet analysis. (G) GO pathway enrichment analysis for predicted target genes expressed in myeloid cells. (H) Schematic model depicting the potential mechanism of TME remodeling driven by interactions among MDSCs, CAFs, vECs, and tumor cells during the malignant progression of BCa by Figdraw (www.figdraw.com). vEC, vascular endothelial cell.

Building on the striking co‐localization of POSTN+ mCAFs and Mono_TYMP, we next applied NicheNet to explore potential cell–cell communication mechanisms. Our analysis revealed that Mono_TYMP cells exhibited heightened activity of ligands, including TGFB1, ITGB1, ITGAM, and VEGFA. TGFB1 binds to TGFBR1/2 and ACVRL1 receptors on POSTN+ mCAFs, while ITGAM ligands interact with ICAM1/ICAM2 and JAM3 receptors, thereby regulating ECM remodeling, connective tissue growth, and angiogenesis (Figure 6D,E, Figure S7J). Additionally, POSTN+ mCAFs, through high expression of TGFB1 and CXCL9, engage FCN1, TGFBR1/R2, CCR1, and C5AR1 receptors on Mono_TYMP, triggering downstream activation of genes involved in regulating inflammatory responses, myeloid cell differentiation, and migration (Figure 6F,G, Figure S7K). In summary, the POSTN+ mCAFs and Mono_TYMP subclusters likely form a reciprocal signaling network that plays a critical role in ECM remodeling and immune suppression in BCa.

### 2.6 Therapeutic targeting of integrin α5β1 potentiates immunotherapy in BCa models

Using single‐cell and spatial transcriptomics, we mapped intercellular communication within the BCa TME, identifying integrin α5β1 as a central mediator of immune–stromal crosstalk and a potential therapeutic target (Figure 6H). Notably, analysis of the BLCA‐TCGA cohort revealed a significant positive correlation between integrin α5β1 and *COL4A1/2* mRNA expression (Figure S8C). Consistent with this, virtual knockout of integrin α5β1 triggered marked transcriptional shifts in genes involved in angiogenesis and vascular remodeling, including *SPARC, COL4A1/2*, *LAMA4*, *LOXL2*, and *VIM* (Figure S8D,E). GO enrichment analysis highlighted pathways related to endothelial migration and differentiation, sprouting angiogenesis, VEGF and integrin signaling, and TGF‐β response, underscoring the pivotal role of integrin α5β1 in orchestrating tumor vascularization (Figure S8F). Functional validation via THP‐1/HUVEC co‐culture assays showed that conditioned medium derived from THP‐1 cells pretreated with RGD peptide, an inhibitor of integrin α5β1–ligand interactions [43], markedly impaired HUVEC migration and tube formation, as indicated by reduced network complexity and decreased crystal violet staining (Figure 7A,B). To further evaluate the therapeutic relevance of integrin α5β1 blockade, we employed an orthotopic BCa model with four treatment arms: control (G1), RGD peptide (G2), anti‐PD‐L1 (Durvalumab, G3), and combination therapy (RGD peptide + anti‐PD‐L1, G4) (Figure 7C). RGD peptide treatment alone significantly reduced tumor volume and weight (Figure 7D,E), with the combination group exhibiting the most pronounced tumor suppression. IF staining showed a marked decrease in VEGFA and CD44 expression following RGD peptide administration (Figure 7F), while qRT‐PCR analysis confirmed a significant downregulation of pro‐angiogenic genes (*CD31*, *VEGFA*, and *ANGPT2*), accompanied by reduced expression of the COL4A–SDC1 collagen signaling axis and the stemness marker *CD44* (Figure 7G). IHC staining for CD31 further demonstrated reduced microvessel density across treatment groups (Figure 7H). These findings collectively suggest that integrin α5β1 blockade suppresses angiogenesis and tumor progression, while potentiating the therapeutic efficacy of ICIs in BCa.

**Figure 7:**
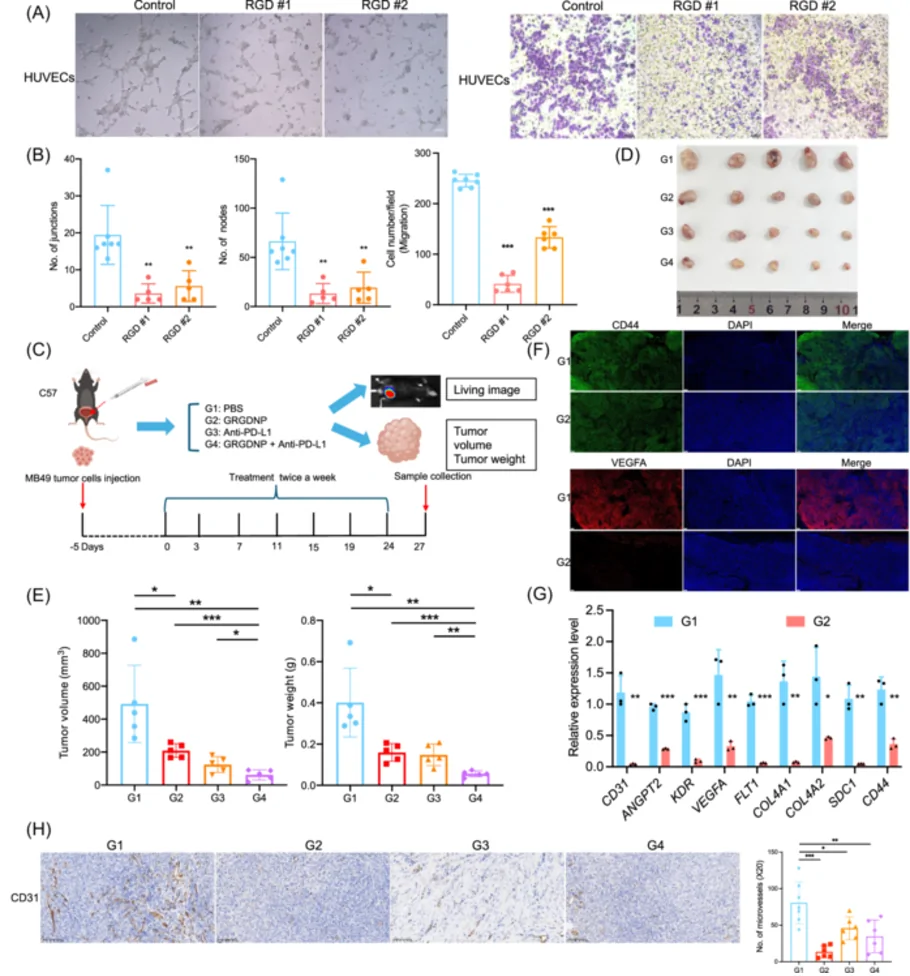
Integrin α5β1 targeting synergizes with immunotherapy in murine models of BCa. (A) Integrin α5β1 blockade impairs endothelial cell tube formation and migration in a co‐culture system. Scale bar = 50 μm. (B) Quantification of in vitro assays (*n* ≥ 5). (C) Experimental design to evaluate the in vivo impact of RGD peptide on tumor growth. (D) Representative images of resected tumors. (E) Quantification of tumor volume and weight at the study endpoint (*n* = 5). (F) IF staining displaying the expression of VEGFA and CD44. Scale bar = 100 μm. (G) Expression of angiogenesis‐ and stemness‐related genes determined by qRT‐PCR. (H) IHC staining evaluating microvessel density. Scale bar = 100 μm. Quantification is shown (*n* ≥ 6). Data are presented as mean ± SD. *p* values were calculated via Student's *t*‐test. *p < 0.05; **p < 0.01; ***p < 0.001. IF, immunofluorescence; IHC, immunohistochemistry.

### 2.7 RGD peptide hydrogel system enhances broad anti‐tumor immunotherapy efficacy

To reduce systemic toxicity and adverse effects associated with conventional drug delivery, we developed a localized drug delivery system by combining alginate (ALG) hydrogel with RGD peptide and anti‐PD‐L1 antibody [44], enabling sustained release and targeted modulation of tumor vasculature and immunity. This system was administered via intratumoral injection in orthotopic BCa models, where the ALG hydrogel rapidly formed a gel at the tumor site to ensure prolonged local retention and controlled release of therapeutic agents. Weekly in vivo imaging revealed significant tumor suppression by the RGD peptide hydrogel system compared to control and ALG‐only groups (Figure 8A–E). The ALG + RGD + PD‐L1 combination group exhibited superior anti‐tumor efficacy, with complete tumor regression in some mice and the slowest decline in overall survival. Flow cytometry revealed a marked reduction in vECs in both the ALG + RGD and ALG + RGD + PD‐L1 groups compared to the ALG group (Figure 8F). Furthermore, the proportions of MDSCs and CD44⁺/CD24⁻ stem‐like tumor cells were significantly reduced in the treatment groups compared to both the control and ALG groups (Figure 8G,H). These findings indicate that the RGD peptide hydrogel system likely suppresses tumor progression by inhibiting angiogenesis, alleviating immune suppression, and depleting stem‐like tumor cells. To explore its applicability beyond BCa, we evaluated the RGD peptide hydrogel system in CT26 colorectal cancer models, including orthotopic and distal tumor settings (Figure 8I). Tumor suppression was evident in both models, with the addition of the anti‐PD‐L1 antibody further amplifying the therapeutic response. Notably, intratumoral injection nearly completely inhibited tumor growth compared to intravenous injection (Figure 8J). Additionally, at later stages, mice receiving intravenous injection showed significantly reduced body weights compared to those treated with intratumoral injection (Figure S9). Collectively, these findings highlight the potential of the RGD peptide hydrogel system in enhancing immune‐based therapies and broadening its application to diverse cancers.

**Figure 8:**
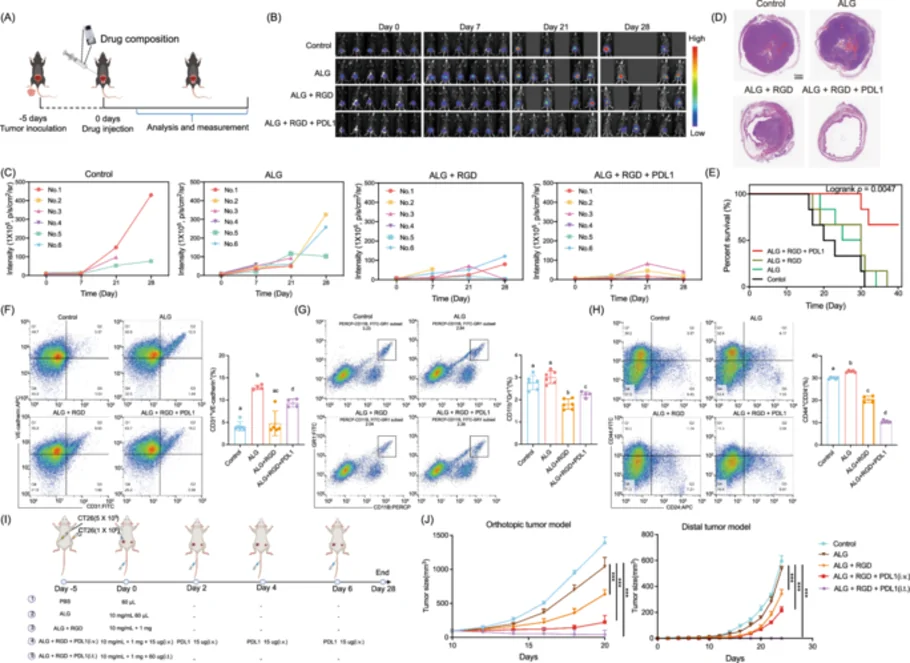
Synergistic enhancement of tumor immunotherapy efficacy through RGD peptide‐integrated hydrogel delivery. (A) Schematic protocol evaluating the therapeutic effect in the orthotopic bladder mouse model. Representative bioluminescence images (B) and quantification of bioluminescence intensity (C) of Luc+ MB49 tumor‐bearing C57BL/6 mice across different treatment groups (*n* = 6). (D) H&E staining of tumor tissues from different treatment groups. Scale bar = 1 mm. (E) Kaplan–Meier survival analysis of tumor‐bearing mice in different treatment groups (*n* = 6, log‐rank *p* = 0.0047). Flow cytometry assay and quantitative analysis of tumor‐infiltrated vECs (F), MDSCs (G), and cancer stem‐like cells (H) after different treatments (*n* ≥ 4). Different letters indicate significant differences (*p* < 0.05, one‐way ANOVA, Tukey's HSD test). (I) Schematic treatment regimen in the CT26 colorectal cancer model. (J) Tumor growth kinetics in both orthotopic (left) and distal (right) colorectal cancer models across different treatment groups. Curves were compared using two‐way repeated measures ANOVA. Data are presented as mean ± SD (*n* = 6). ***p < 0.001 for the indicated comparisons at the indicated time points. ALG, alginate; H&E, hematoxylin and eosin; i.t., intratumoral injection; i.v., intravenous injection.

## 3 DISCUSSION

While current research on BCa has predominantly focused on genetic and epigenetic alterations in urothelial cells [45, 46], the dynamic contributions of the TME remain insufficiently characterized [47]. In this study, we integrated scRNA‐seq and ST‐seq data from both in‐house and public datasets, complemented by IHC, mIF, and animal model validations, to systematically delineate the dynamic remodeling of the TME during BCa progression at single‐cell and spatial resolution. We identified extensive crosstalk between POSTN+ mCAFs and Mono_TYMP, and uncovered their coordinated interactions with vECs, as well as the reciprocal communication between Tip_vECs and stemness‐like tumor cells (Figure 6H). These interactions collectively establish a pro‐angiogenic and immunosuppressive microenvironment, promoting BCa malignant transformation.

CAFs are increasingly recognized as key orchestrators of tumor progression, shaping the microenvironment through pro‐angiogenic, immunosuppressive, and therapy‐resistant programs. Previous spatial profiling in hepatocellular carcinoma (HCC) revealed POSTN+ CAFs orchestrating immunoregulatory niches via IL‐6/IL‐6R signaling with SPP1+ macrophages and interacting with FOLR2+ TAMs and PLVAP+ endothelial cells [48, 49]. Our study extends and mechanistically expands these findings to BCa, demonstrating marked accumulation of POSTN+ mCAFs in high‐grade tumors, spatially adjacent to vECs (Figure 2L) and associated with poor prognosis (Figure 2E). Unlike prior studies, we identify FN1 secretion from POSTN+ mCAFs engaging integrin α5β1 on tip endothelial cells to drive neovascularization (Figures 2K–M, 4G), while concurrently limiting T/NK cell infiltration through MDSC‐mediated immunosuppression (Figure 5F). We further identified TYMP‐high monocytes (Mono_TYMP), transcriptionally resembling early‐stage MDSCs (Figure 3G, Figure S3H), as the predominant myeloid subset co‐localized with POSTN+ mCAFs (Figures 3D, 5E–G,L). These cells engage tip endothelial cells through VEGF and NAMPT–integrin α5β1 signaling (Figures 3, 4) and express high levels of immune checkpoint ligands (Figure 3H,K), consistent with an immunosuppressive phenotype. Regulatory network analysis identified STAT3 [50, 51] and CEBPB as key transcriptional drivers of this program (Figure 3O, Figure S3N,O), with CEBPB linked to canonical pro‐angiogenic effectors such as *VEGFA*, *RHOB*, and *NAMPT* (Figure 3P,Q). Collectively, these data reveal a previously uncharacterized fibro‐myeloid–endothelial axis that coordinates vascular remodeling and immune evasion in progressive BCa, highlighting a mechanistic paradigm distinct from prior CAF‐centric studies.

Angiogenesis is a hallmark of malignant transformation, with tumor endothelial cells (TECs) delivering oxygen and nutrients to support tumor growth and metastasis [52, 53]. Recent studies have highlighted substantial molecular heterogeneity among TEC subsets, particularly angiogenic tip cells, which function as leading regulators of neovascular sprouting through context‐dependent mechanisms [54]. For example, CXCR4+ tip cells and pro‐inflammatory SELR+ venous endothelial cells have been linked to resistance to anti‐angiogenic therapy [38], while APLN+ tip cells and PGF+ tip cells are associated with early neovascularization, epithelial plasticity, and poor prognosis across multiple cancer types [17, 55]. Building on this emerging framework, our study identifies a previously unrecognized population of tip endothelial cells in high‐grade BCa, characterized by COL*4A1*, *KDR*, and *ESM1* expression (Figure 4B). These cells exhibit transcriptional and spatial features consistent with leading‐edge sensors during angiogenic sprouting and respond dynamically to environmental cues, predominantly via VEGFA signaling [56]. Notably, we observed selective enrichment of integrin α5β1 (*ITGA5* and *ITGB1*) on tip endothelial cells across multiple cancer types (Figure 4K), suggesting their potential utility as novel and robust markers for this subset. Furthermore, despite the stromal dilution effect in bulk tissues (Figure S10A), high *ITGA5/ITGB1* levels robustly predict poor prognosis across BLCA, CRC, and GC (Figure S10B–D). Crucially, this broad expression pattern and clinical relevance imply that the specific fibro‐myeloid–endothelial interplay mediated by integrin α5β1 is likely a conserved mechanism across solid tumors, extending the relevance of our findings beyond BCa. Beyond serving as a marker, this selective expression indicates that integrin α5β1 functions as a key signaling node within the CAF/MDSC–vEC communication axis and may represent a tractable therapeutic point of intervention. Building on this observation, we demonstrate that M‐MDSCs and POSTN+ mCAFs promote angiogenesis by engaging tip endothelial cells via the NAMPT/FN1–integrin α5β1 axis (Figure 4G,H). While the downstream signaling cascades remain to be fully elucidated, our findings establish integrin α5β1 as a central conduit linking stromal and myeloid cues to angiogenic activation. Importantly, beyond driving angiogenic sprouting, tip endothelial cells also sustain tumor stemness via the COL4A1/2–SDC1 signaling axis (Figures 1, 6, 7, 8), highlighting their multifaceted regulatory roles within the TME. Clinically, elevated *SDC1* expression and COL4A1/2–SDC1 signature scores significantly correlate with poor prognosis (Figure S10E–G). This interaction exemplifies a multicellular signaling relay, in which sequential ligand–receptor interactions coordinate responses across distinct cellular compartments. Emerging tools such as CellNEST [57] leverage deep learning to decode spatial signaling at single‐cell resolution, providing new opportunities to explore complex intercellular communication in high‐grade BCa. Nevertheless, technical biases in publicly available datasets remain a challenge. Limitations—including sample size imbalance and reduced sensitivity for rare cell types—can compromise data quality even with stringent inclusion criteria. Integrative frameworks such as LIGER [58], CellHint [59], and the Iniquitate pipeline [60] help preserve biological heterogeneity and improve batch correction during data integration.

Despite modest successes of anti‐angiogenic therapies in select malignancies, durable responses remain elusive, largely due to insufficient biomarkers for patient stratification and the complex heterogeneity of the tumor vasculature [38, 61]. Although integrin‐mediated ECM signaling (e.g., α2β1, α1β1, αvβ3, αvβ5) has long been implicated in angiogenesis [62, 63, 64], these approaches generally lack endothelial subtype specificity. In this study, we identify integrin α5β1 as a functionally restricted angiogenic target selectively enriched on tip endothelial cells. Targeting integrin α5β1 with RGD peptides robustly suppressed neovascularization and tumor growth in BCa models, an effect further enhanced by immune checkpoint blockade (Figure 7D–H), distinguishing this strategy from prior integrin‐targeted approaches. To overcome systemic toxicity and heterogeneous drug distribution associated with conventional anti‐angiogenic therapy, we employed ALG hydrogel—a hydrophilic polysaccharide matrix with excellent biocompatibility and responsiveness to the TME—for localized, sustained release [44, 65]. The RGD peptide/anti‐PD‐L1/ALG in situ gel induced significant tumor regression despite an increased abundance of vECs (Figure 8F), consistent with vascular remodeling or normalization rather than vessel depletion [66, 67, 68]. Similar low‐dose effects have been reported for RGD mimetics such as cilengitide [69]. Importantly, the comparable efficacy in CRC models suggests broader translational potential of this approach (Figure 8J). Given the molecular heterogeneity of TECs across tumor types, future studies incorporating comprehensive endothelial atlases, immunocompetent patient‐derived organoids, and humanized patient‐derived xenografts will be essential to refine integrin α5β1‐targeted therapies and facilitate clinical translation [70, 71].

## 4 CONCLUSION

In conclusion, this study integrates scRNA‐seq and ST‐seq to comprehensively characterize the dynamic evolution of the TME during BCa progression. We reveal complex, bidirectional communication among immune cells, stromal cells, and tumor cells, establishing a multidimensional cellular interaction network. In high‐grade BCa, POSTN+ mCAFs and Mono_TYMP cooperatively foster an immunosuppressive and pro‐angiogenic microenvironment. Mechanistically, POSTN+ mCAFs and MDSCs secrete FN1 and NAMPT, respectively, which engage integrin α5β1 on tip endothelial cells, facilitating neovascularization. These Tip_vECs subsequently sustain tumor cell stemness through the downstream COL4A1/2–SDC1 signaling axis. Targeting integrin α5β1 enhances ICI efficacy, with further benefit achieved via ALG hydrogel delivery. These findings suggest integrin α5β1‐mediated stromal signaling may represent a conserved mechanism driving angiogenesis and tumor stemness across malignancies, offering a broadly applicable target with strong translational potential.

## 5 METHODS

### 5.1 Source and processing of clinical samples

Clinical data pertaining to all patients within the single‐cell RNA sequencing (scRNA‐seq) and spatial transcriptomics (ST‐seq) cohorts have been meticulously summarized in the supplementary materials (Figure S1B). Samples in the internal cohort for scRNA‐seq were procured from bladder disease patients who underwent transurethral resection at the Shenzhen Luohu District People's Hospital, while the tumor specimens for ST‐seq were obtained from cases of radical cystectomy. All procedures were conducted in accordance with the ethical guidelines established by the hospital's ethics committee, with written informed consent obtained from each participant (Approval No. 2024‐GDSKJ‐07). Fresh tissue specimens designated for scRNA‐seq were preserved in GEXSCOPETM tissue preservation solution (Singleron, Cologne, Germany) immediately following surgical excision. These samples underwent thorough washing with PBS, were meticulously sectioned into 1–2 mm fragments, and were subsequently subjected to enzymatic digestion utilizing GEXSCOPETM tissue dissociation solution (Singleron). Following digestion, the resulting mixture was filtered through a 40 μm sterile mesh and centrifuged at 1000 rpm for 5 min at 4°C to remove the supernatant. The resultant cell pellet was then resuspended in 1 mL of PBS and treated with 2 mL of GEXSCOPETM red blood cell lysis buffer (Singleron), allowing for a 10‐min incubation on ice to facilitate the lysis of red blood cells. Ultimately, the collected single‐cell suspension was resuspended in PBS in preparation for subsequent scRNA‐seq analysis. Fresh tumor samples for ST‐seq were subjected to rapid freezing through immersion in a mixture of isopentane and liquid nitrogen, subsequently embedded in Optimal Cutting Temperature compound, and stored at −80°C for preservation.

### 5.2 Retrieval and preprocessing of public datasets

We utilized a comprehensive scRNA‐seq dataset comprising unprocessed FASTQ data, stored in the SRA database under BioProject PRJNA662018 [19]. Our analysis focused on samples from 2 cases of low‐grade bladder cancer (BCa), 3 cases of high‐grade BCa, and 1 case of normal tissue. The scRNA‐seq data were meticulously processed and aligned to the human genome GRCh38 using CellRanger (V.3.0.1, 10× Genomics). In addition, we procured processed scRNA‐seq data encompassing 4 cases of adenomatous cystitis, 2 cases of low‐grade BCa,4 cases of high‐grade BCa, and 2 normal samples from the Gene Expression Omnibus (GEO) (GSE135337 [72], GSE222315 [15], GSE225190 [73]). Furthermore, bulk RNA‐seq and microarray data were acquired from two independent cohorts: TCGA‐BLCA (*n* = 406) and GSE31684 (*n* = 93). Lastly, we integrated pan‐cancer scRNA‐seq datasets from GSE161529 (Breast Cancer, BRCA), GSE188711 (Colorectal Cancer, CRC), GSE163558 (Gastric Cancer, GC), and GSE189357 (Lung Adenocarcinoma, LUAD). The integration of these diverse datasets was accompanied by correction for batch effects, accomplished using the R package Harmony (V.0.1.1).

### 5.3 Single‐cell RNA sequencing and functional profiling

Single‐cell suspensions were captured using the GEXSCOPER microfluidic chip, generating gel beads in emulsion. Following cDNA processing and PCR amplification, sequencing was performed on the Illumina HiSeq X platform (Illumina) to obtain 150‐bp paired‐end reads. The raw gene expression matrix was generated using the CeleScope single‐cell data processing software (https://github.com/singleron-RD/CeleScope), with quality control conducted using FASTQC (V.0.11.7) and Cutadapt (V.1.17). Subsequently, data were aligned to the GRCh38 reference genome using STAR (V.2.6.1b), and gene expression matrices were output using FeatureCounts (V.1.6.2). Downstream analysis was conducted using the Seurat R package (V.4.4.0), which included cell filtering and normalization of the count matrix. The Harmony algorithm was applied to eliminate batch effects, which effectively removed technical differences between batches while preserving biological variation [74, 75, 76]. The top 2000 highly variable genes were then selected for principal component analysis (PCA). The FindNeighbors and FindClusters functions were utilized for cell clustering, followed by visualization using the uniform manifold approximation and projection (UMAP) algorithm. Furthermore, cell type annotations for different clusters were then performed using classical marker genes: epithelial cells (*EPCAM*, *KRT13*, *KRT18*, and *KRT7*), myeloid cells (*LYZ*, *CD14*, *FCN1*, *CD68*, *C1QB*, *CD163*, *CD1E*, and *LAMP3*), fibroblasts (*COL1A1*, *COL3A1*, *DCN*, and *FGF7*), T/NK cells (*CD3D*, *CD3E*, *CD2*, *CD4*, *CD8A*, *KLRB1*, and *NCR1*), B cells (*MS4A1*, *CD79A*, *CD79B*, and *CD19*), plasma cells (*MZB1*, *IGHG1*, *IGLC2*, and *IGGKC*), and endothelial cells (*PECAM1*, *PLVAP*, *VWF*, and *CLDN5*). Differential gene expression (DEG) analysis between clusters was performed using the FindAllMarkers function with parameters set to min.pct = 0.25, logfc.threshold = 0.75, and only.pos = TRUE. The non‐parametric Wilcoxon rank‐sum test was used to calculate *p*‐values. These *p*‐values were adjusted using the Benjamini–Hochberg procedure to control the false discovery rate (FDR), with significance defined as an FDR‐adjusted *p* < 0.05 (or *q*‐value < 0.05). Genes with significant differences [FDR‐adjusted *p* < 0.05 and log_2_(fold change) > 0.5] underwent Gene Ontology (GO) enrichment analysis using the clusterProfiler package (V.3.18.1), and significantly enriched biological processes were determined using a threshold of FDR < 0.05. Additionally, pathway activity across cell subclusters was assessed through GSVA on individual cells, calculating average gene expression levels for each subcluster and applying the GSVA package (V.1.38.2) with standard settings. The R package irGSEA (V.1.1.2) was used for gene set enrichment analysis, retrieving relevant gene sets from the msigdbr package (V.7.5.1). For both GSVA and irGSEA, differential pathway activities were statistically evaluated, and significance was established using an FDR < 0.05. Furthermore, the AddModuleScore function from the Seurat package was utilized to assign gene set scores, including the stemness score and the monocytic MDSCs (M‐MDSCs) signature, for each cell subtype. The stemness‐related genes included *CD44*, *ALDH1A1*, *SOX2*, *NANOG*, *OCT4*, *BMI1*, *ABCG2*, and *NOTCH1*, while the MDSC‐associated signature genes consisted of *CD14*, *CD33, ITGAM, S100A8*, *S100A9*, and *STAT3*. The activity of 14 classical pathways in endothelial cell subclusters was assessed using PROGENy (V.1.28.0) [77], with scores calculated via the run_aucell function from the decoupleR package (V.2.12.0), based on the PROGENy network model.

### 5.4 InferCNV analysis

To identify malignant tumor cells, we analyzed CNVs in each epithelial cell subcluster using InferCNV (V.1.6.0). Specifically, we randomly selected 5000 epithelial cells for this analysis, designating T/NK cells and fibroblasts as reference populations to establish a baseline for genomic alterations. A gene ordering file was generated based on the human GRCh38 assembly, detailing the chromosomal positions of each gene. We applied a cutoff of 0.1 for the minimum average read counts per gene in the reference cells and subsequently denoised the output to enhance the accuracy of CNV level predictions.

### 5.5 Cell–cell communication analysis

To evaluate potential cell–cell communication modules among cancer cells, stromal cells, and myeloid cells, we conducted a comprehensive analysis utilizing CellChat (V.1.1.3). Adhering to established protocols, we employed the default CellChatDB.human as our ligand–receptor database to identify overexpressed ligands and receptors within specific cellular subsets, thereby facilitating the inference of cell‐type‐specific communication. Furthermore, we leveraged the updated CellChatv2 (V.2.1.2) to delineate spatially proximal intercellular communications among interacting cell populations derived from spatial transcriptomics. Enhanced ligand–receptor interactions were identified when either the ligand or receptor exhibited upregulation. We utilized the computeCommunProb and computeCommunProbPathway functions to infer the interaction networks of each ligand–receptor pair and their corresponding signaling pathways, which were subsequently visualized through hierarchical plots, circular plots, and heatmaps. The statistical significance of these inferred communications was evaluated based on empirical *p*‐values calculated via the default permutation test, with significant interactions defined as *p* < 0.05.

Concurrently, we performed cell–cell communication analyses between myeloid cells and T/NK cells using the CellPhoneDB Python package [78]. This method leverages a publicly accessible repository of curated ligands and receptors, along with their interaction data, to predict enriched ligand–receptor pairs based on receptor expression in one cell type and corresponding ligand expression in another. By default, only ligands and receptors expressed in at least 10% of cells within each cell type were included in the analysis. Significant pairs were selected based on an empirical *p*‐value < 0.05 (derived from the default permutation test) and a mean expression value ≥ 1 for subsequent evaluations.

Lastly, we employed the NicheNet R package (V.1.1.0) to investigate the signaling mediators involved in ligand‐receptor interactions between fibroblasts and myeloid cells. For both sender and receiver populations, genes of interest within the signaling pathways were required to be expressed in at least 10% of cells for further analysis. In accordance with the NicheNet documentation, the results of this analysis enabled the ranking of potential ligands based on their regulatory potential scores, inference of receptors, and prediction of the primary target genes for these ligands.

### 5.6 Analysis of TF regulons

The R package SCENIC (V.1.3.1) was employed to infer activated regulons across distinct myeloid cell subtypes, utilizing an expression matrix in conjunction with phenotype data. Co‐expression modules were constructed by leveraging the motif database (https://resources.aertslab.org/cistarget/) in conjunction with GRNBoost and RcisTarget to accurately identify relevant regulons. The activity of each regulon within individual cells was assessed through the AUCell function. Following the scaling of the same regulon across different clusters, the results were visualized in a comprehensive heatmap. Additionally, the calcRSS function was utilized to pinpoint specific regulons characteristic of each cell subpopulation.

### 5.7 Trajectory analysis

Trajectory analysis was conducted using Monocle2 (V.2.18.0) [79] to elucidate the developmental processes and lineage differentiation of cancer and myeloid cells. Following established protocols, we imported integrated gene expression data into Monocle2 for cell ordering based on DEGs, with statistical significance rigorously defined at a *q*‐value < 0.01 to control the FDR. Dimensionality reduction was performed using the reduceDimension function with the DDRTree method, limited to two components for enhanced clarity. Subsequently, the differentialGeneTest function was employed to identify genes significantly correlated with pseudotime transitions (*q*‐value < 0.01). To investigate the expression profiles of stemness‐associated genes along differentiation trajectories, we utilized the plot_pseudotime_heatmap function. Specific gene dynamics during differentiation were visualized using the plot_genes_in_pseudotime function. Additionally, we applied CytoTRACE2 (V.1.0.0), a relative scoring method based on the incorporated datasets, to assess cellular developmental potential [80]. This analysis adhered to the recommended default settings, facilitating the estimation of the pseudo‐temporal starting point of cancer cells.

### 5.8 Cell type deconvolution for RNA‐seq datasets

To elucidate the functional roles of distinct cell types within extensive sample compendiums, we employed CIBERSORTx [81] or robust cell‐type deconvolution. This analysis commenced with the generation of a reference signature matrix derived from our single‐cell RNA‐seq dataset, enabling the estimation of cell‐type proportions across publicly available bulk RNA‐seq and microarray datasets. In configuring CIBERSORTx, we disabled quartile normalization for RNA‐seq datasets while enabling it for microarray datasets, retaining all other parameters at their defaults. For imputing cell fractions, we applied a consistent normalization strategy and set the permutation parameter to 500 to enhance result robustness.

To assess correlations among the proportions of infiltrating cell types, we performed Spearman's correlation analysis, visualizing the outcomes using the corrplot package (V.0.90). Furthermore, we utilized the maxstat.test function from the R package maxstat (V.0.7.25) to categorize samples into high and low signature score groups based on an optimal cutoff. The hazard ratio (HR) was calculated using the Cox proportional hazards model with the survival package (V.3.7.0), yielding a 95% confidence interval. Finally, Kaplan–Meier survival analyses were conducted, with statistical significance evaluated through the log‐rank test, elucidating the prognostic implications of cell‐type infiltration.

### 5.9 Tissue handling and data processing for spatial transcriptomics

Embedded OCT tissues were cryosectioned and affixed to Visium Spatial slides (10× Genomics), ensuring an RNA Integrity Number (RIN) of ≥7. Methanol fixation and hematoxylin‐eosin staining were performed to determine the optimal permeabilization time. Subsequently, RT Master Mix was added for reverse transcription, generating spatially barcoded full‐length cDNA, which was then subjected to second‐strand synthesis. The cDNA was amplified via PCR to yield sufficient material for library construction, which involved enzymatic fragmentation and size selection. The libraries were ultimately sequenced on the Illumina NovaSeq. 600 platform (Illumina) in 150 PE mode. Preliminary quality control was conducted on the FASTQ files, followed by genome alignment using the SpaceRanger software (V.1.0.0, 10× Genomics). The ST data were processed using the Seurat package (V.4.4.0). Each sample's gene‐spot matrix was normalized using SCTransform, followed by dimensionality reduction with PCA. Clustering analysis was conducted using the Louvain algorithm on the reduced data, employing 30 PCs and a resolution parameter set to 0.3. UMAP was then utilized for two‐dimensional visualization of the clusters.

### 5.10 Differential expression and pathway enrichment in spatial transcriptomics

DEGs within each cluster were identified through the Wilcoxon rank‐sum test, with genes meeting the criteria of an FDR‐adjusted *p* < 0.05 and log_2_(fold change) > 0.25 considered as candidate marker genes. The functional enrichment analysis of ST data was conducted using methodologies analogous to those employed for scRNA‐seq, with statistical significance maintained at an FDR < 0.05. Signature scores derived from the scRNA‐seq data were subsequently incorporated into the ST dataset's metadata using Seurat's AddModuleScore function with its default settings. To visualize spatial expression patterns, we employed the SpatialFeaturePlot function from the Seurat package, facilitating the generation of spatially resolved feature plots.

### 5.11 Integrated analysis of scRNA‐seq and spatial transcriptomics

We integrated scRNA‐seq and ST datasets using MIA [82]. Cell‐type‐specific gene sets were identified from scRNA‐seq data, and spatially specific gene sets from ST data. The overlap between these gene sets was measured to assess enrichment or depletion, with significance evaluated using *p*‐values from the hypergeometric distribution. Cell type distributions across spatial contexts were analyzed, and cell cluster depletion was quantified using −log10(1 − *p*). Moreover, we employed the CellTrek package (V.0.0.94), leveraging the Delaunay triangulation approach [83], to spatially co‐embed the scRNA‐seq data and analyze co‐localization patterns among various cell types. We generated a sparse graph with a random forest model to create a spot‐cell similarity matrix, then summarized co‐localization patterns using the scoloc function. The strength of cell co‐expression was evaluated with Kullback–Leibler divergence (KLD), where higher KLD values indicated stronger co‐localization of cell subclusters.

### 5.12 Virtual knockout of integrin α5β1 using scTenifoldKnk

To investigate the regulatory role of integrin α5β1 (ITGA5/ITGB1), we performed gene knockouts using the scTenifoldKnk R package (V.1.0.2) [84]. Count matrices were extracted from Seurat‐processed scRNA‐seq data, and highly variable genes were selected for network construction. Simulated knockouts were conducted with quality control filters (mitochondrial content < 10%, library size > 1000), generating 10 subsampled networks (500 cells each) and using 3 principal components. Differential regulation was quantified by Euclidean distance, Box‐Cox–normalized Z‐scores (*Z*), and fold changes. Significance was determined by |*Z* | > 2 and FDR‐adjusted *p* < 0.01 (Benjamini–Hochberg). Visualization of top‐ranked genes revealed marked transcriptional perturbations following *ITGA5* and *ITGB1* knockout, highlighting the critical role of integrin α5β1 in orchestrating tumor–stromal and immune interactions.

### 5.13 IHC, IF, and mIF staining

Formalin‐fixed, paraffin‐embedded tissue sections (5 μm) were dewaxed and rehydrated on slides. For immunohistochemistry (IHC), antigen retrieval was performed by heating sections in 100°C citrate buffer for 15 min. Endogenous peroxidase activity was inhibited using 3% hydrogen peroxide for 20 min. To prevent nonspecific binding, sections were incubated with 5% normal goat serum at room temperature for 1 h, followed by overnight incubation at 4°C with the primary antibody. After washing with PBS, sections were incubated at room temperature with HRP‐conjugated secondary antibody for 1 h, then stained with DAB and counterstained with hematoxylin. Images were acquired using K‐Viewer software (V.1.0.2). The antibodies used included anti‐CD31, anti‐HIF1A, and anti‐SDC1 (all from Servicebio, China). For immunofluorescence (IF) and multiplex immunofluorescence (mIF), antigen retrieval was performed by heating sections in 0.1 M sodium citrate buffer (pH 6.0) at 95–99°C for 10 min. After washing, sections were incubated with 10% goat serum in PBS for 30 min to block nonspecific binding, followed by overnight incubation at 4°C with primary antibodies conjugated to distinct fluorophores. Afterward, HRP‐conjugated secondary antibodies were applied, and tyramide signal amplification was conducted. Sections were microwaved after each staining cycle, and nuclei were stained with DAPI (GDP1024, Servicebio). The following antibodies were employed in the study: anti‐COL3A1, anti‐CD31, anti‐FN1, anti‐ITGB1, anti‐CD11b, anti‐VEGFA, anti‐NAMPT, and anti‐CD44 (all from Servicebio, China), as well as anti‐POSTN (ab14041, Abcam). Image acquisition was performed using CaseViewer 2.4 (3DHISTECH). To assess the average distances between NAMPT‐positive, FN1‐positive, and ITGB1‐positive cells, spatial distance correlation analysis was conducted on the mIF scan results using the HALO platform (V.3.0.311.314) from Indica Labs.

### 5.14 qRT‐PCR assays

Total RNA was isolated from the samples using TRIzol reagent (Invitrogen), followed by precipitation with isopropyl alcohol. The quality and concentration of theextracted RNA were assessed using a NanoDrop spectrophotometer (NanoDrop). For cDNA synthesis, a commercial kit (Vazyme) was employed, following the provided protocols. mRNA levels were quantified through qRT‐PCR using SYBR Green‐based kits (Vazyme). The expression of target genes was normalized to *GAPDH* in each sample. The primer sequences utilized for qRT‐PCR are as follows: *GAPDH* (forward, 5′‐CCAGCAAGGACACTGAGCAA‐3′; reverse, 5′‐GCCCCTCCTGTTATTATGGGG‐3′), *CD31* (forward, 5′‐CCGATTCCTGGATTGCGAGA‐3′; reverse, 5′‐AGCGCCTCTGAGTCTCTGTA‐3′), *CD44* (forward, 5′‐TGCAAGTACTCCACGGGTTT‐3′; reverse, 5′‐TGGTTCCGGGGTCCAATTTT‐3′), *SOX4* (forward, 5′‐GACCTGAACCCCAGCTCAAA‐3′; reverse, 5′‐AGCCGGGCTCGAAGTTAAAA‐3′), *ALDH1A1* (forward, 5′‐TAGCTGATGCCGACTTGGAC‐3′; reverse, 5′‐TCTTAGCCCGCTCAACACTC‐3′), *KDR* (forward, 5′‐AGATGCATTGTGCTGGCTCT‐3′; reverse, 5′‐GCCTGTAACCCGACTGTGAA‐3′), *FLT1* (forward, 5′‐TCAAGGACGGCTTTGCAGAT‐3′; reverse, 5′‐TGGAGTTCGGTGAAAGCTCC‐3′), *ANGPT2* (forward, 5′‐CTACTCGCTCAAGGCCACAA‐3′; reverse, 5′‐TGTGGAAAGGGGCTGCTATG‐3′), *VEGFA* (forward, 5′‐TTCGGGAACCAGACCTCTCA‐3′; reverse, 5′‐GACCCAAAGTGCTCCTCGAA‐3′), *SDC1* (forward, 5′‐TTTGGGGTCGCTGAAGTTGT‐3′; reverse, 5′‐AGAGTCATCCCCAGAGCCAT‐3′), *COL4A1* (forward, 5′‐TTTCGACTTGCGGCTCAAAG‐3′; reverse, 5′‐ ACTGCCTGGGAATCCAACTC‐3′), *COL4A2* (forward, 5′‐CTCTAGCCAAACCCACAGCA‐3′; reverse, 5′‐TGACACAGGTTGTAGGACGC‐3′), *CEBPB* (forward, 5′‐AGAAGACCGTGGACAAGCAC‐3′; reverse, 5′‐ GCTTGAACAAGTTCCGCAGG‐3′), *NAMPT* (forward, 5′‐ CCAATCACAGTGGCCACAAAT‐3′; reverse, 5′‐ CTCCTATGCCAGCAGTCTCT‐3′), *RHOB* (forward, 5′‐ CAGGTGGAGCTGGCGCTGT‐3′; reverse, 5′‐ CACCAGGATGATGGGCACAT‐3′), *NECTIN2* (forward, 5′‐ TTCGTCTGCACAGTCACCAA‐3′; reverse, 5′‐ CAGCAGTAGCAATGATGGCG‐3′).

### 5.15 Cellular function assays

To elucidate the role of SDC1 in tumor cells, targeted knockdown of SDC1 was achieved through RNA interference (RNAi) utilizing Lipofectamine™ RNAiMAX Transfection Reagent (Invitrogen). The efficiency of knockdown was quantitatively assessed via qRT‐PCR and western blot analysis using a monoclonal anti‐Syndecan 1 antibody (HY‐P81745, MCE; 1:2000). For spheroid formation assays, BCa cell lines (UMUC‐3) with or without SDC1 knockdown were seeded in six‐well plates pre‐coated with 2% poly (2‐hydroxyethyl methacrylate) (P3932, Sigma‐Aldrich) at a density of 20,000 cells per well. The cells were cultured in a non‐adherent environment using DMEM‐F12 medium (D6434, Sigma‐Aldrich), augmented with 1× B27 (1:50, 17504044, Invitrogen), 20 ng/mL EGF (AF‐100‐15, PeproTech), and 4 µg/mL insulin (11376497001, Roche), and treated with human collagen IV (Corning, Cat. No. 354245) at 20 µg/mL at each passage. After three passages, images were captured and analyzed using MoticamX (Motic Europe S.L.U., Barcelona, Spain). For cell proliferation, the Cell Counting Kit‐8 (CCK8) assay kit (Dojindo) was employed to evaluate CCK8 activity. Specifically, 2000 cells were seeded into 96‐well plates and incubated for 24 h. Subsequently, 10 μL of CCK‐8 solution was added to each well, followed by an additional 3‐h incubation. Absorbance was measured at 450 nm using a microplate reader (RT‐6200, Rayto, USA). In the wound‐healing assay, tumor cells were seeded in six‐well plates and cultured until they reached 90% confluence. A sterile 200 μL pipette tip was utilized to create an artificial wound within the monolayer. Images of the wounded area were captured at both 0 and 60 h using an inverted microscope (100×, Nikon Corporation).

To evaluate the role of integrin α5β1 in endothelial function, HUVECs (ATCC® CRL‐1730™) and THP‐1 monocytes (ATCC® TIB‐202™) were co‐cultured in Transwell® systems (Corning®). For tube formation assays, THP‐1 cells (5 × 10³) were seeded in the upper chamber (0.4 μm pore size) with 10% FBS DMEM, while HUVECs (1 × 10⁵) were plated on Matrigel‐coated 24‐well plates (40 μL/well, polymerized at 37°C) and treated with conditioned medium (CM) derived from THP‐1 cells pretreated with RGD peptide (100 μM, MCE) or PBS. After 6 h of incubation, tubular structures were imaged (Carl Zeiss, AG) and quantified using the Angiogenesis Analyzer plugin in ImageJ based on junction and node metrics. For migration assays, HUVECs (1 × 10⁵) in serum‐free DMEM were seeded in the upper chamber of 8 μm Transwell® inserts, while the lower chambers contained THP‐1 cells in 10% FBS DMEM with or without RGD peptide (100 μM). After 12 h, migrated HUVECs were fixed with 4% paraformaldehyde, stained with 0.1% crystal violet, and quantified in randomly selected fields using ImageJ.

To verify the functional role of the mCAF–FN1 ligand in mediating stromal‐vascular crosstalk, supernatant transfer experiments were performed using human bladder stromal fibroblasts (HBdSF, passage 3) that were serum‐starved overnight upon reaching 70–80% confluence. Cells were treated for 24 h in serum‐free medium with PBS, 20 ng/mL recombinant human FN1 (rFN1; Cat. No. 4305‐FNB, R&D Systems, Minneapolis, USA), 1.0 µg/mL FN1 functional blocking antibody (FN1 ab; Cat. No. LS‐C83489, LSBio), or a combination of rFN1 and FN1ab. To isolate paracrine signaling and eliminate residual exogenous agents, cells were washed three times with sterile PBS and cultured in fresh serum‐free medium for another 24 h; supernatants were then collected, centrifuged at 2000 × *g* for 10 min to remove debris, and mixed 1:1 with DMEM containing 10% fetal bovine serum (FBS). This mixed conditioned medium was subsequently utilized as a chemoattractant in the lower chamber of Transwell systems for HUVEC migration assays or used to resuspend HUVECs for Matrigel‐based angiogenesis tube formation assays, with migration counts and tube metrics (junctions and nodes) quantified according to standard protocols.

Finally, to investigate the specific contribution of MDSCs to angiogenesis, M‐MDSCs were differentiated from healthy donor PBMCs (isolated via Ficoll gradient) by culturing for 7 days in RPMI‐1640 supplemented with GM‐CSF and IL‐6 (20 ng/mL each; PeproTech). Monocytic MDSCs (CD11b⁺CD33⁺HLA‐DR^low) were sorted by flow cytometry (BD Biosciences) and transfected with CEBPB siRNA (HY‐RS02446, MCE). The knockdown efficiency of CEBPB and the expression of downstream targets (*NAMPT*, *RHOB*, and *NECTIN2*) were assessed by RT‐qPCR post‐transfection. Functionally, conditioned medium from CEBPB‐silenced M‐MDSCs was applied to HUVECs (1 × 10⁵) to evaluate endothelial sprouting. Tube formation was quantified (nodes/junctions) 6 h after seeding on Matrigel, while migration was assessed using Transwell inserts (12 h incubation), followed by fixation, crystal violet staining, and counting via ImageJ.

### 5.16 Animal model studies

All experimental mice were housed in specific pathogen‐free conditions, with approval from the Institutional Animal Care and Use Committee (IACUC) of Shenzhen Following Precision Medical Research Institute (Approval No. 20240408002). Six to eight‐week‐old C57BL/6 mice underwent orthotopic bladder wall injection of Mb49 tumor cells (1 × 10^6^) and were randomized into four treatment groups (5–8 mice per group) 5 days post‐injection: PBS, 6 mg/kg RGD peptide (GRGDNP, MCE), 0.5 mg/kg anti‐PD‐L1 (Durvalumab, MCE), and a combination therapy of RGD peptide and anti‐PD‐L1, administered intraperitoneally twice weekly. For local treatment in the orthotopic bladder tumor model, MB49 tumor‐bearing mice were randomly assigned to four treatment groups (*n* = 6 per group): PBS, ALG (10 mg/mL; 60 µL), ALG + RGD peptide (10 mg/mL + 1 mg), and ALG + RGD peptide + anti‐PD‐L1 (10 mg/mL + 1 mg + 60 µg). After inoculating MB49 tumor cells for 5 days. Subsequently, each group received a single intratumoral injection of the respective treatment directly into the bladder tumor following surgical exposure. Throughout the treatment period, tumor growth was monitored via a multimodal in vivo imaging system (AniView100, BioLight, Guangzhou, China), with photographic documentation. At the experimental endpoint, tumors were excised for volumetric and weight assessments, followed by formalin fixation and paraffin embedding for subsequent IHC and IF staining, and RNA was extracted for qRT‐PCR. For flow cytometry, tumor tissues were minced and digested in RPMI1640 at 37°C with collagenase IV (1 mg/mL, Sigma) and DNase I (1 mg/mL, Roche) for 1 h. The resultant cell suspension was filtered through a 70 μm cell strainer, subjected to red blood cell lysis, and incubated with fluorescently labeled antibodies at 4°C for 20 min, followed by two washes with cold PBS. Flow cytometry was performed utilizing a BD FACSCanto II Flow Cytometer (BD Biosciences), with analysis performed using FlowJo software (V.10.7.1, LLC, Ashland, OR). The antibodies employed included PerCP Anti‐Mouse CD11b (ab91145, Abcam), FITC Anti‐Mouse Ly‐6G/Ly‐6C (FITC‐65140, Proteintech), PE Anti‐Mouse CD45 (PE‐65087, Proteintech), FITC Plus Anti‐Mouse CD31 (FITC‐65058, Proteintech), APC Anti‐Mouse VE‐cadherin (#138012, BioLegend), FITC Anti‐Mouse CD44 (ab30405, Abcam), and APC Anti‐Mouse CD24(ab239286, Abcam).

For the CT26 distal and orthotopic colorectal cancer mouse models, 1 × 10^5^ and 5 × 10^5^ CT26 cell suspensions were injected into the distal colon and the colon/rectum, respectively. After 5 days of tumor establishment, the CT26 tumor‐bearing mice were randomly divided into five groups. Treatment schedules and dosages are as indicated in Figure 8I. Tumor volumes and the body weights of the mice were measured every other day.

### 5.17 Statistical analysis

Data analysis was performed using R software (V.4.0.3) and GraphPad Prism software (V.8.1.2). Survival curves were estimated using the Kaplan–Meier method and compared via the log‐rank test, with groups defined by median or optimal cutoff values. Spearman correlation analysis was employed to assess relationships between variables. For continuous variables, differences between two groups were evaluated using the Student's *t*‐test or the Wilcoxon rank‐sum test, as appropriate. For multiple group comparisons, one‐way ANOVA followed by Tukey's HSD test was used for parametric data. Two‐way ANOVA with Dunnett's test was applied for experiments involving two independent factors, whereas two‐way repeated measures ANOVA was employed for longitudinal data with multiple time points. To control the false discovery rate in non‐parametric multiple comparisons, *p*‐values derived from the Wilcoxon rank‐sum test were adjusted using the Benjamini–Hochberg method. A two‐tailed *p* < 0.05 was considered statistically significant.

## AUTHOR CONTRIBUTIONS

**Ting Liang, Tao Tao**, and **Song Wu**: Conceptualization; supervision. **Ting Liang, Wuwu Xu**, and **Hu Fang**: Methodology; software; formal analysis; data curation. **Lu Fu**: Data curation; formal analysis; investigation. **Dongdong Tang** and **Xing Xiong**: Resources; clinical sample collection. **Dashi Deng, Chenchen Li, Lisha Liu**, and **Huancheng Yang**: Investigation. **Ting Liang, Wuwu Xu, Hu Fang, Lu Fu**, and **Fang Liu**: Validation. **Ting Liang, Wuwu Xu, Hu Fang, Guangzhi Li**, and **Song Wu**: Funding acquisition. **Ting Liang, Tao Tao, Guangzhi Li**, and **Wuwu Xu**: Visualization; writing—original draft. **Guangzhi Li, Tao Tao**, and **Song Wu**: Writing—review & editing. **Tao Tao**: Project administration. All authors have read the final manuscript and approved it for publication.

## CONFLICT OF INTEREST STATEMENT

The authors declare no conflicts of interest.

## AI STATEMENT

The preliminary version of this manuscript underwent grammar refinement and sentence structure optimization with the assistance of Gemini 3.1 Pro (https://gemini.google.com/). All content generated by the AI tool was thoroughly reviewed and revised by the authors to guarantee the scientific accuracy of facts and eliminate any potential biases. We hereby affirm that no AI tools were utilized in the procedures of raw data generation, downstream bioinformatics analysis, or the rendering of final visual content.

## ETHICS STATEMENT

Bladder tumor samples were obtained from patients undergoing transurethral resection of bladder tumors at Shenzhen Luohu District People's Hospital. Written informed consent was obtained from all participants, and the study protocol was approved by the hospital's Institutional Review Board (IRB) (Approval No. 2024‐GDSKJ‐07). All animal procedures complied with the ARRIVE Guidelines and were approved by the Institutional Animal Care and Use Committee (IACUC) of Shenzhen Following Precision Medical Research Institute (Approval No. 20240408002). Mice were housed under specific pathogen‐free (SPF) conditions with *ad libitum* access to autoclaved food and water.

## Supporting information


**Figure S1:** Single‐cell expression profiling and functional characterization of epithelial cells during BCa progression.
**Figure S2:** Fibroblast cluster annotation and functional characterization.
**Figure S3:** Characterization of myeloid cells during BCa progression.
**Figure S4:** CEBPB is required for the pro‐angiogenic function of M‐MDSCs.
**Figure S5:** The characteristics and alterations of endothelial cells during BCa progression.
**Figure S6:** Spatial architectural differences in low‐grade versus high‐grade bladder tumors.
**Figure S7:** Spatial disparities in cell communication between high‐grade and low‐grade tumors.
**Figure S8:** Integrin α5β1 blockade disrupts the COL4A–stemness axis and inhibits tumorigenesis.
**Figure S9:** Body weight changes in orthotopic (left) and distal (right) colorectal cancer models.
**Figure S10:** Clinical relevance of the COL4A1/2–SDC1 axis and integrin α5β1 signaling in BCa and pan‐cancer datasets.

## Data Availability

The data that support the findings of this study are available from the corresponding author upon reasonable request. The data generated in this study have been deposited in the OMIX database of the China National Center for Bioinformation (CNCB) under the accession number OMIX015404 (https://ngdc.cncb.ac.cn/omix/preview/6lcfgVxv). Previously published public datasets analyzed in this study are described in detail in the METHODS section. Due to ethical and legal restrictions related to human participant data, deidentified individual‐level data and associated clinical metadata cannot be made publicly available. All data are available upon reasonable request to the corresponding author and subject to institutional policies and local rules and regulations. All customized analytical pipelines and R scripts used in this study are publicly available in the GitHub repository at https://github.com/liangting19930920-bit/BCa-scRNA-ST-Analysis-2025. Supplementary materials (figures, graphical abstract, slides, videos, Chinese translated version, and updated materials) could be found in the online DOI or iMetaOmics http://www.imeta.science/imetaomics/.
