# Supplementary material for: Integrin α5β1‐mediated multicellular crosstalk in the tumor microenvironment drives bladder cancer progression and reveals targetable vulnerabilities
Source: IMetaOmics. 2026 Jun 23;3(2):e70102. doi: 10.1002/imo2.70102 (PMC13674414; doi:10.1002/imo2.70102)
Supplement: Supplementary file 1 [file IMO2-3-e70102-s001.docx]

**Supporting information to**

**Integrin α5β1-mediated multicellular crosstalk in the tumor microenvironment drives bladder cancer progression and reveals targetable vulnerabilities**

**Running title**: Integrin α5β1 drives TME crosstalk in bladder cancer

Ting Liang^1#^, Wuwu Xu^2,3#^, Hu Fang^4#^, Lu Fu^2,3#^, Dashi Deng^2,3^, Chenchen Li^2,4^, Lisha Liu^5^, Dongdong Tang^6^, Huancheng Yang^2^, Fang Liu^1^, Xing Xiong^2^, Guangzhi Li^2,3*^, Song Wu^1,2,3*^, and Tao Tao^2,3*^

^1^College of Electronics and Information Engineering, Shenzhen University, Shenzhen 518060, China.

^2^Department of Urological Surgery, The Third Affiliated Hospital of Shenzhen University (Shenzhen Luohu People's Hospital), Shenzhen University, Shenzhen 518116, China.

^3^Shenzhen Following Precision Medical Research Institute, Luohu Hospital Group, Shenzhen 518000, China.

^4^Institute of Biomedical Data, South China Hospital, Medical School, Shenzhen University, Shenzhen 518116, China.

^5^Institute of Pain, The Affiliated Hospital of Southwest Jiaotong University, The Chengdu Third People's Hospital, Chengdu 610014, China.

^6^Department of Urology, The First Affiliated Hospital of Zhengzhou University, Zhengzhou 450052, China.

^#^These authors contributed equally: Ting Liang, Wuwu Xu, Hu Fang, Lu Fu

*Correspondence: [taotao@zxbiomed.org](mailto:taotao@zxbiomed.org) (Tao Tao); [wusong@szu.edu.cn](mailto:wusong@szu.edu.cn) (Song Wu) ; guangzhili@email.szu.edu.cn (Guangzhi Li).

**Figure S1 Single-cell expression profiling and functional characterization of epithelial cells during BCa progression**. (A) UMAP plot displaying the distribution of 24 tissue samples. (B) Summary of all sample information. (C) Total cell numbers of each cell type are shown. (D) Proportions of seven major cell types are shown in bar plots in different sample tissues. (E) UMAP plots of epithelial cells, displaying clustering patterns (top) and distribution across sample types (bottom). (F) Bar plots showing epithelial cell subcluster proportions across sample types. (G) UMAP plot showing CNV levels in epithelial cells. (H) Copy number variations in epithelial cells, inferred by InferCNV, with T/NK cells and fibroblasts as references. (I) Top 10 differentially upregulated genes in distinct epithelial cell subclusters. (J) Heatmap displaying the top 15 differentially upregulated genes in epithelial cells across distinct sample types. (K) GSVA analysis of epithelial cells across distinct sample types. (L) Immunohistochemical images of SDC1 and HIF1A across tumor progression stages. Scale bars = 200 μm. (M) The pseudotime expression of *SDC1* and *HIF1A*. (N) Pseudotime analysis of transcriptional changes in selected genes across different sample types from epithelial cells. (O) Stemness scoring across different sample types from epithelial cells. *p* values were calculated via two-sided Wilcoxon rank-sum test; ****p* < 0.001. (P) The correlation between the expression of *SDC1* and stemness signature in TCGA-BLCA cohort. (Q) The effect of SDC1 on cell migration in UMUC3 cells, assessed by wound-healing assay, with statistical analysis of the migration rate shown on the right (n ≥ 3). Scale bar = 200 µm. Statistical analysis by Student’s *t*-test; ****p* < 0.001.

**Figure S2 Fibroblast cluster annotation and functional characterization. (A) UMAP plots displaying the subclusters of fibroblasts.** (B) Violin plots showing the expression of marker genes across different subclusters. (C) The correlation between angiogenesis and *POSTN* gene expression. (D) High levels of *POSTN* predict poor prognosis in the TCGA-BLCA cohort. (E) Kaplan-Meier overall survival curves stratified by CCL11+ iCAF infiltration in TCGA-BLCA patients. (F) Mountain plot illustrating the enrichment scores of angiogenesis-related gene sets across different sample types in fibroblasts. (G) GSVA analysis across different CAF subtypes. (H) GO term enrichment across different CAF subtypes. (I) Heatmap displaying the top 15 differentially upregulated genes in fibroblasts across distinct sample types. (J) Circle plots depicting differences in the number and strength of cellular interactions between low-grade and high-grade tumors. (K) Top representative differentially expressed ligand–receptor signaling pathways between low-grade and high-grade tumors. (L) Representative IF staining of low-grade and high-grade tumor tissues showing DAPI (blue), POSTN (green), COL3A1 and FN1 (red), and CD31 and ITGB1 (yellow). Scale bar = 50 µm.

**Figure S3 Characterization of myeloid cells during BCa progression. (A) UMAP plots displaying the subclusters of myeloid cells**. (B) Violin plots illustrating marker gene expression across myeloid cell subclusters. (C) Volcano plot illustrating the expression of the top five most significantly upregulated and downregulated genes among the myeloid cell subtypes. (D) Correlation between angiogenesis pathway scores and *TYMP* expression. (E) Pearson correlation between the signature scores of the Mono_TYMP and mCAF_POSTN subtypes. (F) The absolute infiltration levels of Mono_TYMP in low-grade and high-grade tumor samples. (G) The top 50 pathway enrichment scores per cell across different myeloid cell subsets, analyzed by irGSEA. (H) Vlolin plots illustrating the expression of MDSC-associated marker genes across different myeloid cell subpopulations. (I) CellChat analysis of alterations in signaling interaction strength from myeloid cells to endothelial and T/NK cells in high-grade BCa. (J) Pseudotime analysis of transcriptional changes in functional genes of Mono_TYMP across myeloid cell subtypes. (K) Expression of key ligands (NAMPT and VEGFA) across different myeloid cell subtypes. (L–M) Representative IF staining of low-grade and high-grade tumor tissues displaying DAPI (blue), CD11b (cyan), NAMPT and PD-L1 (green), VEGF (red), and CD31 and ITGB1 (yellow). Scale bar = 100 µm. (N) Heatmap showing the mean activity of the top 10 differentially activated regulons across myeloid cell subtypes, as inferred by PySCENIC. (O) Top5 ranked specific transcription factors in the Mono_TYMP subtype.

**Figure S4** **CEBPB is required for the pro-angiogenic function of M-MDSCs.** (A) Schematic overview of the experimental workflow. M-MDSCs were differentiated from human PBMCs using GM-CSF and IL-6, sorted (CD11b+CD33+HLA-DRlow), and transfected with CEBPB siRNA. The conditioned medium (CM) was collected to treat HUVECs. (B) RT-qPCR verification of CEBPB knockdown efficiency in M-MDSCs transfected with three independent siRNAs (#1, #2, #3) compared to negative control (NC). (C) Relative mRNA expression levels of key functional genes (*NAMPT*, *RHOB*, and *NECTIN2*) in M-MDSCs following CEBPB silencing. (D–E) Representative images (D) and quantitative analysis of tube nodes and junctions (E) from Matrigel tube formation assays. HUVECs were incubated with CM derived from M-MDSCs transfected with NC or CEBPB siRNA. Scale bar = 50 μm. (F–G) Representative images (F) and quantification (G) of HUVEC migration in Transwell assays. Scale bar = 100 μm. Data are presented as mean ± SD. *p* values were calculated via Student's *t*-test. **p* < 0.05, ***p* < 0.01, ****p* < 0.001.

**Figure S5 The characteristics and alterations of endothelial cells during BCa progression.** (A) Heatmap displaying the top 15 differentially upregulated genes in endothelial cells across distinct sample types. (B) GSVA analysis in endothelial cells across distinct sample types. (C) Bubble plot illustrating the expression of angiogenesis-related genes in endothelial cells across different sample types. (D) Expression of *ANGPT2*, *COL4A2*, and *COX2* across different endothelial cell subclusters. (E) UMAP plot illustrating the expression of classical marker genes in endothelial cells. (F) Mountain plot illustrating the enrichment scores of angiogenesis gene sets across various sample types (top) and endothelial cell subtypes (bottom). (G) Expression of key ligand-receptor pairs for *FN1*, *NAMPT*, and *ITGA5*/*ITGB1* across different cell types. (H) Heatmap displaying the interaction flow of VISFATIN and VEGF signaling networks across various myeloid cell subtypes and endothelial cell subsets. (I) Contribution of each ligand-receptor pair in FN1 signaling pathways across various CAF subtypes and endothelial cell subsets. (J) Bubble plot showing signaling pathway interactions from mCAF_POSTN to different endothelial cell subtypes. (K) Correlation between *ITGA5*/*ITGB1* and *PECAM1* expression in the TCGA-BLCA cohort.

**Figure S6** **Spatial architectural differences in low-grade versus high-grade bladder tumors.** (A) UMAP of ST-seq data showing sample origin and seven subclusters. (B) Heatmap of canonical marker gene expression across seven spot clusters. (C) Bubble plot displaying GO enrichment terms across different spatial regions. (D) Heatmap of GSVA results highlighting pathway activity differences across spatial regions. (E) Spatial feature plots illustrating hypoxia signature scores. (F) Spatial feature plots illustrating stemness signature scores. (G) Spatial feature plots illustrating angiogenesis signature scores.

**Figure S7 Spatial disparities in cell communication between high-grade and low-grade tumors.** (A) Heatmaps depicting the results of hypergeometric distribution analysis between cell types identified by scRNA-seq and regions defined by ST analysis. (B) Co-embedding analysis integrating single-cell and spatial transcriptomics using CellTrek in high-grade tumor tissues. (C) Circle plots depicting the interaction frequency and strength across different spatial regions. (D–F, I) Circle plots illustrating the spatial distribution of NAMPT–ITGA5/ITGB1, FN1–ITGA5/ITGB1, VEGF, and COL4A2–SDC1 ligand-receptor pairs, along with violin plots depicting the expression profiles of ligands and receptors across spatial regions. (G–H) Spatial colocalization of the NAMPT–ITGA5/ITGB1 and FN1–ITGA5/ITGB1 ligand-receptor pairs in high-grade tumor tissues. (J) Predicted target genes of fibroblasts regulated by top-ranked ligands in myeloid cells. (K) Top-ranked ligands in fibroblasts and their interacting receptors in myeloid cells, along with UMAP plots of CXCL9 receptor and C5AR1 ligand expression.

**Figure S8** **Integrin α5β1 blockade disrupts the COL4A–stemness axis and inhibits tumorigenesis.** (A) Left: Representative micrographs of colony formation in UMUC3 cells subjected to the indicated treatments. Scale bars = 100 μm. Right: Bar graphs showing the statistical quantification of colony numbers (n ≥ 10). (B) Relative mRNA expression of stemness-associated markers in UMUC3 cells following different treatments, determined by qRT-PCR. Data are presented as mean ± SD. (C) Spearman correlation analysis showing a significant positive association between integrin α5β1 (or *ITGA5*/*ITGB1*) and *COL4A1/2* mRNA expression in the TCGA-BLCA cohort. (D) Horizontal bar plot showing the top 20 perturbed genes following virtual KO of ITGA5/ITGB1; FC indicates fold change relative to baseline regulation. (E) Volcano plot displaying global regulatory shifts upon KO, with thresholds marked at –log10(0.05) for FDR-adjusted *p*-values and Z = ±2. (F) GO enrichment analysis of the top 500 differentially regulated genes post-KO.

**Figure S9 Body weight changes in orthotopic (left) and distal (right) colorectal cancer models.** Curves were compared using two-way repeated measures ANOVA. Data are shown as mean ± SD (n = 6). ****p* < 0.001 for the indicated comparisons at the indicated time points. i.v., intravenous; i.t., intratumoral.

**Figure S10** **Clinical relevance of the COL4A1/2–SDC1 axis and integrin α5β1 signaling in BCa and pan-cancer datasets**. (A) Pan-cancer expression landscape of integrin α5β1 (derived from *ITGA5* and *ITGB1* levels) in tumor (red) and normal (green) tissues. (B–D) Kaplan–Meier survival analyses of Overall Survival in patients with bladder cancer (BLCA), colorectal cancer (CRC), and gastric cancer (GC), stratified by integrin α5β1 expression. High expression of integrin α5β1 is significantly associated with poor prognosis in all three cohorts (*p* < 0.05). (E) Kaplan–Meier analysis of overall survival (OS) in the TCGA-BLCA cohort stratified by high versus low *COL4A1/2* (*COL4A1* and *COL4A2*) expression levels. (F) Pan-cancer expression profile of *SDC1* in tumor (red) versus matched normal (green) tissues from the TCGA dataset, analyzed using the GEPIA2 platform. (G) Kaplan–Meier survival analysis of TCGA-BLCA patients stratified by the COL4A1/2–SDC1 signature score. The signature was defined as the average expression of *COL4A1*, *COL4A2*, and *SDC1*. Patients were stratified into high- and low-signature groups based on the median expression value.
